# Microbial communities of selected regions of the Deep Springs Lake aquifer system

**DOI:** 10.3389/fmicb.2025.1689006

**Published:** 2025-12-18

**Authors:** Rania Zaki, Emma Bourne, Andrew Storino, Jay Nadeau

**Affiliations:** 1 Deep Springs College, Big Pine, CA, United States; 2 Department of Physics, Portland State University, Portland, OR, United States

**Keywords:** soda lake, Deep Springs Valley, hypersaline, alkaliphiles, microbiome

## Abstract

Deep Springs Lake is a small, isolated, highly alkaline soda lake in Inyo County of Eastern California, USA. It is a seasonally filled salt lake or playa, and is part of a closed aquifer system. Such closed systems are globally rare, occurring only in arid zones where annual evaporation is greater than annual rainfall. Deep Springs Lake’s hydrology and geology have been well studied, and it is home to a unique toad species, but its microbiome remains unexplored. Here we perform 16S, 18S, and ITS amplicon sequencing of the lake water, dried salt crust at the edges the lake, and nearby feeder springs to investigate the community composition of prokaryotes, eukaryotes, and fungi. Bacterial communities in the lake water consist predominantly of Pseudomonadota and Bacteroidota. Nearby springs and salt crust contain different genera of Pseudomonadota than the lake water but similar Bacteroidota, along with an abundant population of Chlorobiota. Noticeably rare in the lake itself but abundant in the biofilms and crust are populations of photosynthetic Cyanobacteria. Archaea are found only in the lake water, largely Halobacterota. Fungi are mostly Ascomycota, with some Chytridiomycota and Rozellomycota; chytrid fungi show no evidence of pathogens related to amphibian die-offs. Eukaryotes in the lake water consist mostly of flagellates, notably the photosynthetic *Dunaliella*, and brine shrimp (*Artemia*). In order to compare these sites with source waters elsewhere in the watershed, we also perform 16S amplicon sequencing of three feeder springs found at higher elevations remote from the lake. The Pseudomonadota found in the remote sites differ from those in the lake at the genus level or higher. Some of the genera of Bacteroidota found in the lake are also seen in the remote springs, while most are unique to the springs. Taxonomy and Bayesian source/sink analysis show that the microbiome of Deep Springs Lake derives very little input from the remote feeder springs, but contains extremophiles similar to those of soda lakes worldwide. Further investigation of the lake and its surrounding springs may lead to the identification of new species of bacteria, fungi, and eukaryotes and allow comparisons with other closed aquifer systems.

## 1 Introduction

Highly alkaline “soda” lakes can be found in a wide variety of climatic zones. They require a basin with limited or absent outflow (usually in volcanic terrain), high evaporation rates (characteristic of arid or semi-arid environments), and low soluble magnesium and calcium concentrations. Multiple soda lakes can be found in the African Rift Valley (Getenet et al., 2023), British Columbia (Paquette et al., 2024), the Carpathian Basin, and the Great Basin and Mojave desert regions of California and Nevada east of the Sierras (Oremland, 2021; Soufi et al., 2024).

There is increasing biotechnological interest in soda lakes for numerous reasons. Enzymes that function at extremes of salinity and pH are useful in laboratory and industrial applications. Soda lake bacteria are capable of degrading or otherwise processing many pollutants, including hydrocarbons and heavy metals such as arsenic, and of generating hydrogen and methane (Haines et al., 2023; Uma et al., 2020; Varshney et al., 2023; Sarwa et al., 2024).

A fundamental understanding of the community composition and diversity of these unique ecosystems can also help shed light on the origin and evolution of life on Earth, as well as help inform the search for microbial life elsewhere in the solar system. Minerals characteristic of soda lakes have been suggested to exist on solar system moons such as Ceres, Europa, and Enceladus (Carrozzo et al., 2018; McCord et al., 1999; Glein et al., 2015), making the question of the link between these environments and the origin of life increasingly interesting. Even more recently, it has been suggested that soda lakes might have served as the cradle for life on Earth because of their ability to concentrate phosphate (Toner and Catling, 2020; Haas et al., 2024; Kempe and Kazmierczak, 2002). Phosphate is a limiting nutrient, and explanations for how it could have been present in sufficient quantities for the origin of life were lacking before this discovery.

One of the best studied soda lakes is Mono Lake in California, which has been shown to harbor several unique species, including brine shrimp, nematodes, the microalga *Picocystis*, and the large choanoflagellate *Barroeca monosierra*, all interacting with and influencing microbial diversity (Stamps et al., 2018; Junkins et al., 2019; Kanzaki et al., 2021; Sainz-Escudero et al., 2021; Hake et al., 2024). Despite the numerous ongoing discoveries in Mono Lake, other soda lakes in California remain little studied. In this study we report preliminary sequencing results from a soda lake for which no microbiome data has previously been reported.

Deep Springs Lake (DSL) is an alkaline lake located in Inyo County of Eastern California, USA at an elevation of 1495 m. The lake is in the southeast corner of the Deep Springs Valley, enclosed by the Inyo and White Mountains. Its hydrology and mineralogy have been extensively studied since the sixties (Jones, 1965). The Deep Springs Valley aquifer system is an isolated and self-contained watershed. Recent fault uplifts occurring after the eruption of the Pleistocene Bishop Tuff (0.772 Ma) hydrologically isolated the valley by blocking streams from flowing out of the basin (Knott et al., 2023). The basin is now surrounded by high mountains (the White and Inyo ranges) that do not permit surface water runoff. Closed aquifer systems such as these are globally rare and occur only in arid and semi-arid zones where annual evaporation is greater than annual rainfall, such as the Quinqhai-Tibet Plateau, the Bitlis Highlands in Turkey, and the Great Basin playa where Deep Springs lake is located (Tomonaga et al., 2017; Tang et al., 2022; Langbein, 1961).

The watershed is mainly fed by two creek systems, the Wyman-Crooked Creek System and the Birch-Antelope Creek System, along with three major spring groups close to the lake: Corral Springs, Buckhorn Springs, and Bog Mound Springs. The Bog Mound Springs, the Wyman-Crooked Creek stream, and the Birch-Antelope Springs are all connected as part of the same aquifer system (Jones, 1965). The Corral Springs appear to release groundwater from igneous rocks in a fault zone to the north of the lake, while the Buckhorn springs discharge what is predominantly groundwater from sedimentary and contact metamorphic rocks. Another major source of water in the aquifer is rainfall. Annual averages between 1.8 and 10 inches per year in the Deep Springs Valley were reported for the period 1948–2006, with an overall annual mean for the period of 6.0 inches (Jones, 1965; Western Regional Climate Center [WRCC]).

Another area of interest in Deep Springs Valley is that it is home to an endangered species of black toad, *Anaxyrus* (formerly *Bufo*) *Exsul*, found only in the springs adjacent to the lake and never more than 12 m from a water source (Wang, 2009; Murphy et al., 2003). Amphibian populations are in decline globally, and a major factor in extinctions is fungal infection by the chytrid *Batrachochytrium dendrobatidis*. Sequencing the toad habitat will reveal the presence of chytrid fungi and of bacteria that influence the species’ cutaneous microbiome and likely determine susceptibility to fungi, as has been seen in frogs in the Sierra Nevada (Lam et al., 2010; Jani and Briggs, 2018).

In this study, we perform 16S, ITS, and 18S rRNA sequencing of DSL lake water and what we term “salt crust,” a dry, white saline mineral assemblage characteristic of the DSL saltpan whose mineralogy has been described in detail (Jones, 1965). We also sample and sequence from Corral Springs immediately adjacent to the lake. 16S sequencing is performed from three feeder sites from more remote areas of the watershed: Wyman Creek and two springs above Wyman Creek. Taxa are assigned and Picrust analysis is used to evaluate metabolic pathways, and Bayesian source-sink analysis is used to evaluate the contributions of the remote and nearby feeder springs to the lake. Results are discussed in the context of what is known about soda lakes worldwide.

## 2 Materials and methods

### 2.1 Sample collection

**FIGURE 1:**
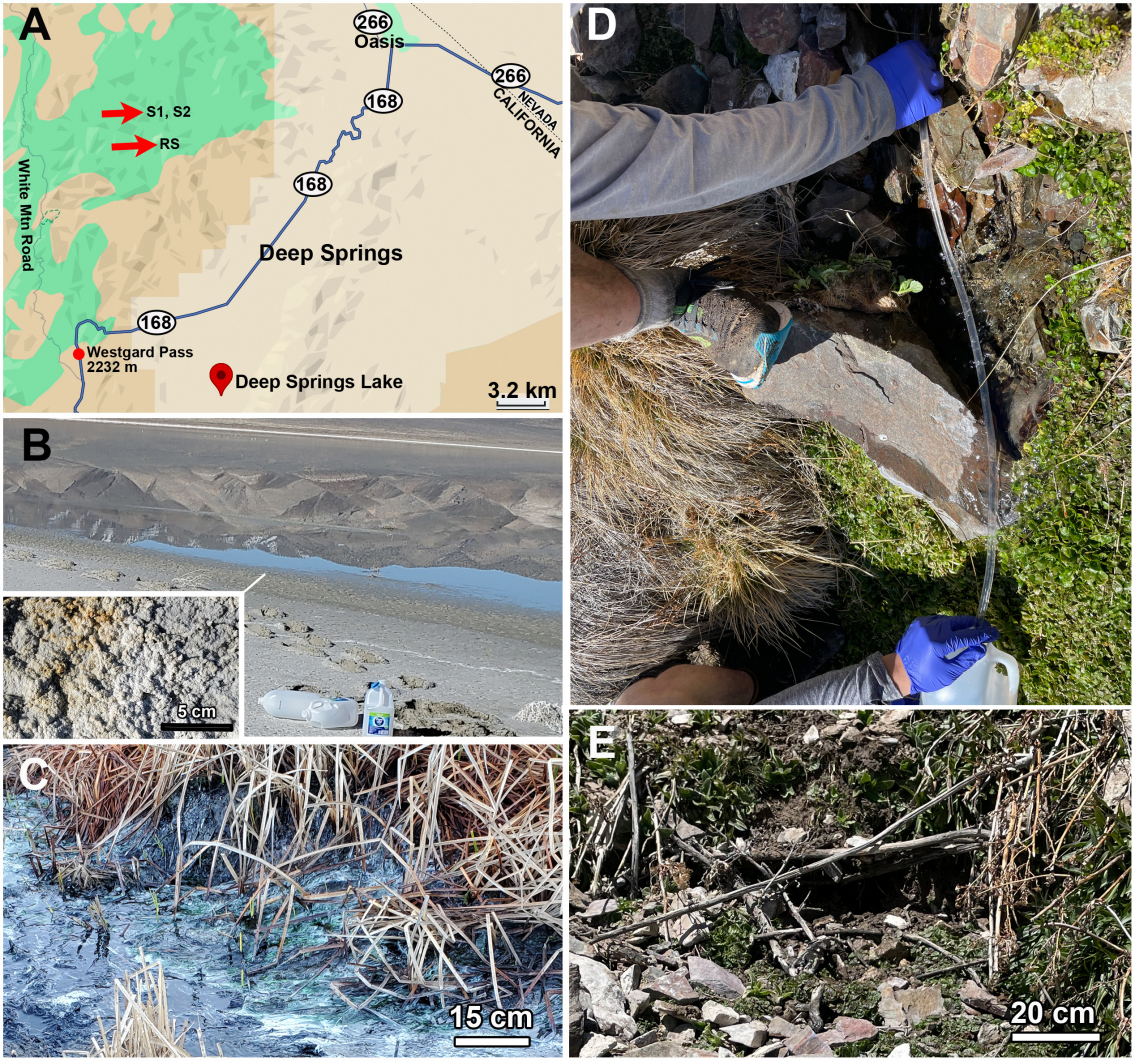
Sampling areas. **(A)** DSL is located near the California/Nevada border just off California Highway 168 several km down from Westgard Pass. The sampled springs, Spring 1, Spring 2 (S1, S2) are located in the mountains directly north of the lake, 1245 m higher in elevation. **(B)** Appearance of DSL sampled area with bottles for scale. The insert shows a closeup of the salt crust. **(C)** Green biofilm mats located in flowing channels from Corral Springs. **(D)** Sampling from Spring 1 by gravity flow using sterile tubing. **(E)** Spring 2. Flow was slower than in Spring 1, requiring ∼1 h to collect 2 L.

Water and salt crust samples were taken from the northeastern edge of Deep Springs Lake on March 20, 2024 (37°16’50.63” N, 118°01’54.11” W) (elevation: 1495 m) (Figures 1A, B, Supplementary Figures 1, 2). Additional samples were collected from a shallow stream to the southeast of the lake emerging from Corral Springs (37°16’26.29” N, 118°01’23.51” W) (Figure 1C). Stream biofilm samples immersed in their surrounding water and dry salt crust samples were collected into sterile Whirl-Pak sampling bags (Sigma Aldrich). Lake water samples were collected using sterile 2 L polyethylene bottles. Samples were returned to the laboratory (∼10 km distant) by motor vehicle and processed in <30 min, except for one bottle of lake water that was deliberately left at room temperature overnight in order to evaluate the importance of immediate extraction. For DNA extraction from water samples, 1–2 L were collected onto a 0.2 μm polyethersulfone Sterivex-GP pressure filter (Sigma-Aldrich) using a Gast DAA-V155-EB vacuum pump through sterilized silicone tubing (Cole-Parmer). For biofilm and salt crust, 0.5 mL of sample was processed for DNA extraction. The filtered water was stored at +4 °C for elemental analysis. pH and electrical conductivity were measured immediately in both raw and filtered water.

On April 3, 2024, three additional remote sites were sampled (Figure 1A), hereafter referred to as Spring 1 (S1) (37°27’10.04” N, 118° 06’ 40.36” W, elevation 2740 m), Spring 2 (S2) (37°27’9.9” N, 118° 06’ 41.51” W, elevation 2740 m), and Roberts Ranch Stream (RS) (37°25’51.02” N, 118° 05’ 58.02” W, elev. 2470 m) (see Supplementary Figures 1–3 for additional maps at higher and lower resolution). Spring 1 and Spring 2 were both located on a hillside above Wyman Creek, along Mill Canyon Stock Trail, and were sampled at the highest elevation of observed surface flow. Care was taken not to disturb or pollute the spring prior to sampling. There was insufficient water at the surface to sample directly, so water was collected into sterile 2 L polyethylene bottles using a 30 cm length of sterile PVC tubing that was inserted into the spring to a depth of 5 cm and allowed to collect into the bottle via gravity flow (Figures 1D, E); the process of collection took ∼30 min for a 1 L volume. Roberts Ranch Spring was a section of Wyman Creek located 270 vertical m below S1 and S2. At this site there was abundant flow of surface water, so a sterile polyethylene bottle was held in the creek for several seconds until filled. Spring and creek samples were transported at ambient outdoor temperatures (∼15 °C) back to the laboratory, a distance of ∼ 15 km. Because of the remote nature of the sites, they were accessed on foot and transport took 2 h. The samples were flash-frozen immediately upon return to the laboratory, then thawed 12 h later and filtered as described above.

Supplementary Table 1 provides a summary of the samples collected.

### 2.2 Trace mineral analysis

Trace elements were quantified by the Soil Health Lab, Oregon State University using an Agilent 5110 Synchronous Vertical Dual View Inductively Coupled Plasma-Optical Emission Spectrometer (ICP-OES). Samples were measured in Radial view at a viewing height of 12 mm. A rinse time of 20 s, uptake time of 15 s, and stabilization time of 12 s was achieved for sample. A 5 ppm standard for all elements of interest was measured as an internal standard. 6 mL of each sample or standard was measured in triplicate.

### 2.3 DNA extraction and sequencing

DNA was extracted immediately after collection (for biofilm/salt crust) or filtration (for water) using a PowerWater DNA kit (Qiagen) according to the manufacturer’s instructions, with the optional high-temperature incubation step performed. A blank sample consisting of a membrane filter processed in the kit alongside the microbial samples was also included. After extraction, DNA was flash-frozen in elution buffer and stored at −20 °C before being shipped on water ice to Molecular Research, Shallowater, TX for sequencing. The same samples were used for bacterial, fungal, and eukaryotic sequencing. Sequencing methods were provided by Molecular Research (see Supplementary material). Bacterial sequencing used the 16S rRNA gene V4 variable region PCR primers 515/806 (Fadeev et al., 2021; Walters et al., 2016); fungal sequencing used the ITS1F/ITS2 primer pair (Usyk et al., 2017); and 18S sequencing used the 1391f/EukBR primers targeting the V9 variable region of the 18S rRNA gene (Choi and Park, 2020).

### 2.4 Data analysis

All 16S and ITS sequence-based analyses were performed in QIIME 2 (Estaki et al., 2020). Raw Illumina reads were demultiplexed by Molecular Research and the provided data were imported into QIIME using the “Casava 1.8 paired-end demultiplexed fastq” option. Primers were removed using cutadapt (Martin, 2011), and data were denoised using the dada2-denoise-paired plug-in to remove low-quality, chimeric, and artifactual sequences (Callahan et al., 2016). These sequences were then dereplicated into amplicon sequence variants (ASVs).

For 16S analysis, taxonomy was assigned to each ASV using QIIME’s feature-classifier plug-in and the Silva 138.2 database (Quast et al., 2013). ASVs assigned to mitochondria, chloroplasts, eukarya, or not otherwise identified as bacterial or archaeal were excluded from further analysis. Alpha diversity indices (Chao1, Shannon) were calculated using QIIME 2. Principal coordinate analysis (PCoA) was visualized using EMPeror (Vázquez-Baeza et al., 2013) within QIIME2 using unweighted and weighted Unifrac, Jaccard, and Bray-Curtis methods (Halko et al., 2011; Hamady et al., 2010; Lozupone and Knight, 2005; Lozupone et al., 2011). See Supplementary Figures 4, 5 for quality plots of forward and reverse reads, Supplementary Table 2 for read counts, and Supplementary Table 3 for denoising stats). For abundance analysis, sequences occurring at 0.2% of total frequency per sample or at <500 total counts across all samples were filtered using “filter features” in QIIME 2.

For ITS, Supplementary Figures 6, 7 show quality plots of forward and reverse reads, Supplementary Table 4 shows read counts, and Supplementary Table 5 gives denoising stats. Taxonomy was assigned using a classifier trained using untrimmed sequences from the UNITE v. 10 database (2024) (Abarenkov et al., 2024).

Eukaryotic data were analyzed using a curated NCBI database and plotted by Molecular Research (see Supplementary material).

Nested bar plots of top taxa were produced in fantaxtic (Teunisse, 2022). Heatmaps were created using the heatmaps feature of QIIME 2.

Functional analysis was performed using Picrust2 (Douglas et al., 2020). The Picrust2 full pipeline was run on the Galaxy EU server (The Galaxy Community, 2024). Parameters used were: minimum alignment length 0.8, predict discrete traits by maximum parsimony, transition cost weight 0.5, maximum nearest-sequenced taxon index (NSTI) 2.0. The output consisted of KO identifier frequencies for each sample, which were exported and converted to KEGG pathways using the KEGG database (Kanehisa, 2002). Analysis of differential expression was performed using Statistical Analysis of Metagenomic Profiles (STAMP) (Parks et al., 2014), with comparison between groups performed using White’s non-parametric two-sided *t*-test.

Source tracking was used to provide estimates of the contribution of each of the springs to the lake. The software employed was SourceTracker (Knights et al., 2011) which uses Gibbs sampling, a Markov chain Monte Carlo algorithm. This approach has been successfully used to analyze 16S OTU distributions from a variety of sources (Liu et al., 2018; Staley et al., 2018; Brown et al., 2019; O’Dea et al., 2019). The probability that a specific sequence will be assigned to a given environment is given by Knights et al. (2011).


$$P\left(z_{i}=v|z^{-i},x\right)\propto P\left(x_{i}|v\right)P\left(v|x^{-i}\right)=\{\begin{array}{c} \frac{m_{x_{i}\,v}+\alpha_{1}}{m_{v}+\tau \,\alpha_{1}}\times \frac{n_{v}^{-i}+\beta }{n-1+\beta \,V}\,v<V\\ \frac{m_{x_{i}\,v}+n\,\alpha_{2}}{m_{v}+\tau \,n\,\alpha_{2}}\times \frac{n_{v}^{-i}+\beta }{n-1+\beta \,V}\,v=V \end{array},$$

where *P*(*t*|*v*) is the probability of a source emitting from a specific taxon *i*, and *P*(*v*) is the probability of a sequence having come from a given source. α_1_ and β are small values added to all OTUs so that their abundance does not start at 0, and α_2_ allows for the creation of an Unknown community by specifying what percent of the total sink sequence count should be added to the Unknown. Sequences in a sink are randomly assigned to a source and iterated to convergence. We used rarefaction depths of 1000 and default values of a and b and varied the number of iterations between 100 and 10000. Tracking was also performed non-negative matrix factorization (NMF) (Huang et al., 2024).

### 2.5 Imaging

Lake, spring, and creek water were concentrated by partially passing 1 mL of sample in a sterile syringe through a 0.22 μm filter to reduce the volume in the syringe by 5- to 10-fold. The concentrated volume remaining in the syringe was then collected into 1.5 mL tubes for imaging. Biofilm segments with their associated water were placed into 1.5 mL tubes and large pieces were dissociated by pipetting. All samples were examined by fluorescence microscopy after staining with acridine orange (Sigma Aldrich) or SYTO9 (Fisher Scientific). Samples were exposed 2–5 min to ∼1 μM acridine orange; samples were then centrifuged and rinsed in 0.9% sterile NaCl and resuspended before spreading onto slides. SYTO9 in a 5.0 mM stock solution in dimethylsulfoxide (DMSO) was first diluted to 500 μM in phosphate-buffered saline (PBS), pH 7.4, then added to 1 mL of sample to a final concentration of 0.5–1.0 μM. The sample was rocked for 1–2 min and then imaged immediately without washing.

Imaging was performed using a portable Thorlabs Cerna as described previously (Case et al., 2024) with 450 nm LED excitation (2118.1 mW minimum LED, M450LP2, Thorlabs) with an auramine longpass filter cube (450/50 excitation; 485 nm dichroic; 495 nm longpass emission) (No. 19008; Chroma, Bellows Falls, VT). The objective lens was a Mitutoyo UV Plan-Apo (20 × NA = 0.5), a Nikon 60 × NA = 0.8, or a Mitutoyo Plan-Apo 100 × NA = 0.7). Images were collected at selected single wavelengths (±10 nm) or as spectral sweeps, both using liquid crystal tunable filter (LCTF) (KURIOS-WB1, 420–730 nm, 35 nm full width half maximum; Thorlabs). An Andor Zyla 4.2 sCMOS (Oxford Instruments) camera was used for all images.

Images were acquired in increments of 10 nm using the Micromanager 2.0 software (Edelstein et al., 2014), and the resulting image stacks were analyzed in Fiji (Schindelin et al., 2012). Illuminant images in HDR format were generated in Scyven (Scyllarus, Canberra, Australia) for hyperspectral images. This results in pseudocoloring the emission bands according to a chosen palette; unless stated otherwise, our hyperspectral images correspond to a “real color” RGB palette.

## 3 Results

### 3.1 Hydrology

The measured pH of the lake was 9.4, and of Corral Springs (where the biofilm was collected) 8.4. In contrast, the springs and Wyman Creek were close to neutral (pH 7.8 in both springs, 7.9 at Wyman Creek/Roberts Ranch Stream). Trace mineral analysis performed on these samples, along with more complete analysis found in previous reports, are tabulated in Supplementary Table 6. In general, the lake water is hypersaline (sodium concentrations 2000–4000 mM and higher), while the Corral Springs water shows variable sodium values from 1.7 to 10 mM and the springs and Wyman Creek are freshwater (sodium levels of 0.3–0.5 mM).

### 3.2 Bacteria and Archaea (16S)

#### 3.2.1 Alpha and beta diversity

The lake water and Corral Springs samples showed similar Chao1 and Shannon diversity indices, while the three remote springs showed significantly greater diversity than either of these, *p* < 0.05 (Supplementary Figures 8, 9). Beta diversity analysis showed distinct clustering of the remote springs, the biofilm and salt crust, and the 3 lake water samples (including the overnight-incubated sample). The beta diversity of the lake water sample left at room temperature overnight diverged from that of the freshly collected samples (Supplementary Figure 10).

#### 3.2.2 Abundances

**FIGURE 2:**
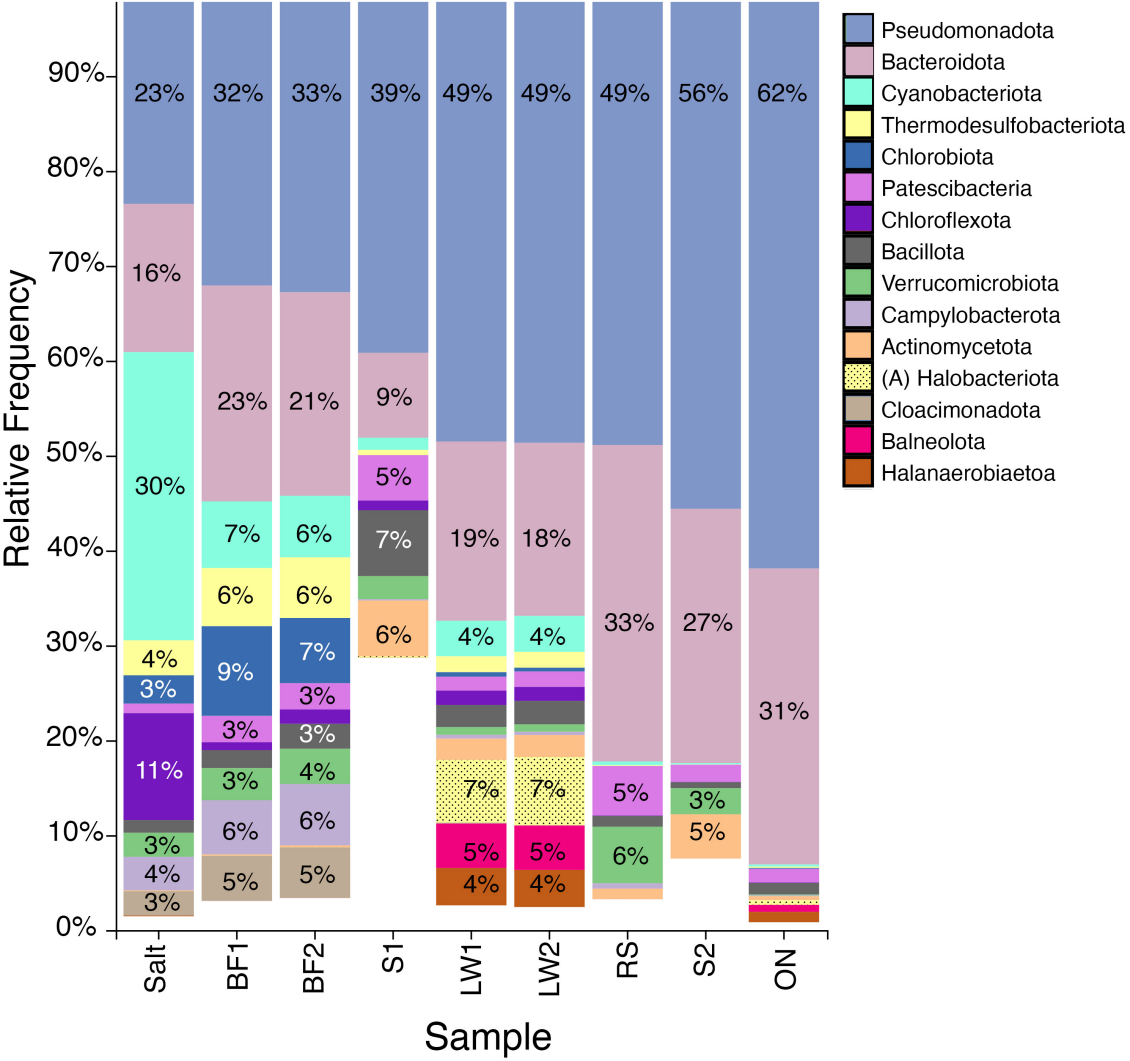
Prokaryotic abundances by phylum in springs (Wyman Creek/Roberts Ranch Stream [RS], Spring 1 [S1], Spring 2 [S2]), lake (LW1, LW2, and overnight incubation [ON]), and biofilm (BF1, BF2, and salt crust [Salt]) for the top 15 phyla across all samples. Samples are filtered by prevalence and ordered by increasing concentrations of Pseudomonadota. Percentages are given for phyla occurring at 3% or more (rounded to the nearest integer). Spring 1 (and to a lesser degree Spring 2) contained several phyla not included in the top 15 overall.

The lake water, at the phylum level, was dominated by Bacteroidota and Pseudomonadota, accounting for nearly 70% of the reads. Other phyla occurring at >4% were Cyanobacteria, Halobacteriota (Archaea), Balneolota, and Halanerobiaeota. Overnight incubation at room temperature (20 °C) led to a notable increase in the Pseudomonadota and a nearly complete disappearance of the Cyanobacteria and Halobacteriota, so that 93% of the overnight-incubated lake water consisted of Pseudomonadota and Bacteroidota. In contrast, the salt crust samples contained only 39% Pseudomonadota/Bacteroidota. The Corral Creek biofilm samples showed similar phyla to the salt crust, though with different relative abundances, most notably a lesser percentage of Cyanobacteria. Both salt crust and biofilm contained abundant populations of Thermodesulfobacteriota, Chlorobiota, Campylobacterota, and Chloroflexota. The remote springs/Wyman Creek contained mostly Pseudomonadota and Bacteroidota, with smaller fractions of Patescibacteria, Verrucomicrobiota, and Actinomycetota (Figure 2).

**FIGURE 3:**
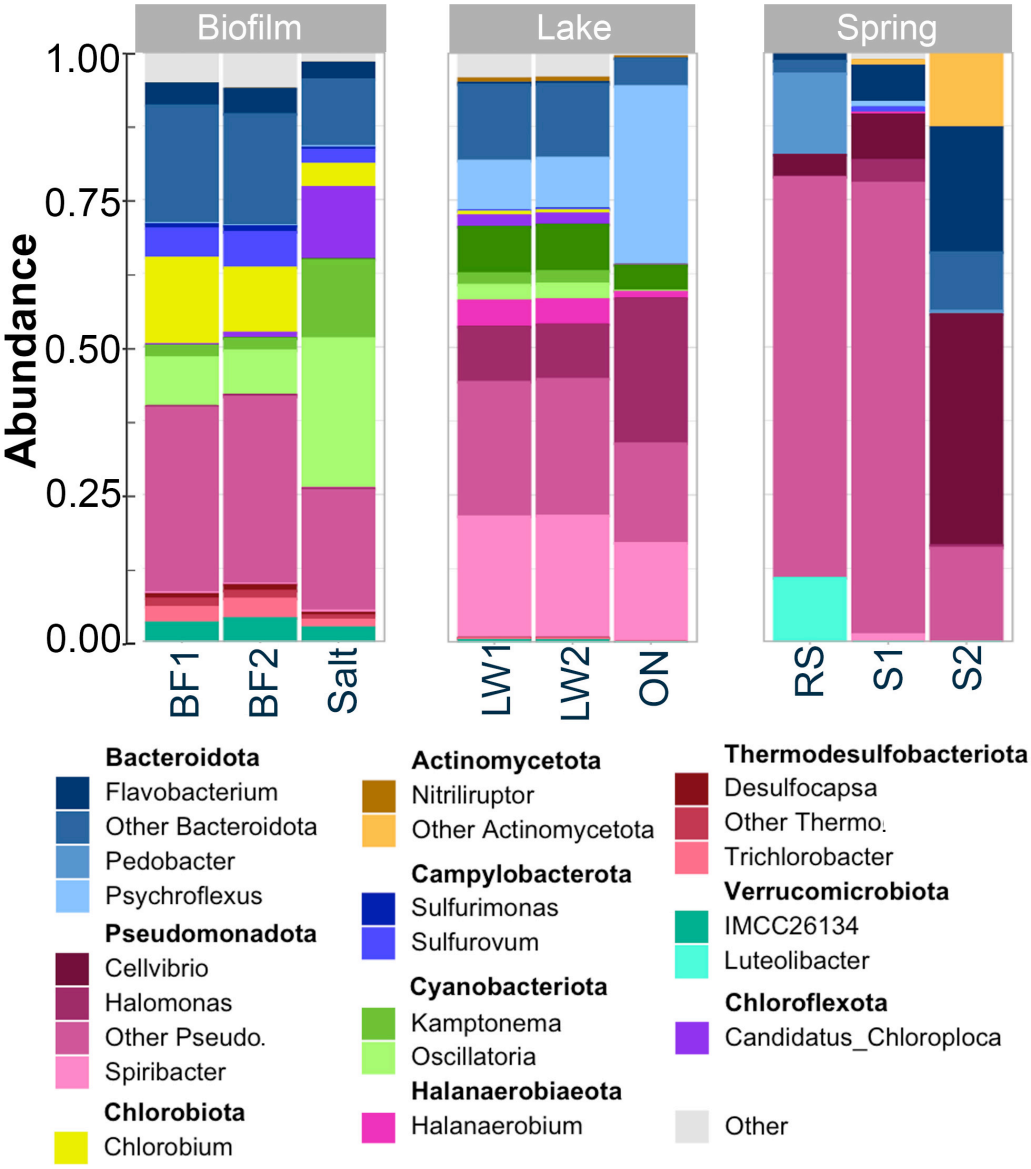
Relative abundances of the top 3–4 genera (or families if not otherwise classified) in the top 10 phyla of bacteria and Archaea in the biofilm (left), lake (center), and springs (right). In all sites, the top 2 phyla were Bacteroidota and Pseudomonadota. Cyanobacteria were present in biofilm and salt crust, and to a lesser extent in lake, while the springs contained no cyanobacteria but an abundant fraction of Actinomycetota. The identity of the 4th most prevalent phylum was different at each site: Chlorobiota in biofilm, Halanaerobiaeota in lake, and Verrocomicrobiota in springs.

Full classification by genus is shown in Supplementary Figure 11. In order to better illustrate the differences between the sites, the top four genera in the top ten phyla are shown in Figure 3, accounting for over 90% of the classified sequences. *Flavobacterium* was one of the top three Bacteroidota in all sites. The lake water contained almost equal proportions of the Bacteroidota *Psychroflexus*, *ML365J_aquatic_group*, and *Flavobacterium*. The springs contained *Flavobacterium*, *Psychroflexus*, and *Pedobacter*.

Within the Pseudomonadota, *Halomonas, Spiribacter*, and *Thioalkalivibrio* dominated the lake water. The biofilm contained the Pseudomonadota *Rugosibacter*, *Thiocapsa*, and *Thiotrix*. Pseudomonadota in the springs and creek were largely *Cellvibrio*, *Halomonas*, and *Sphingomonas*. The third most prevalent phylum in the biofilm was Cyanobacteria, consisting mostly of *Oscillatoria* but with a sizeable number of *Kamptonema*. In the lake water, not including the overnight incubated sample, some cyanobacteria were present. In the springs and creek, only trace cyanobacteria were seen; Actinomycetota were the third most common phylum, largely *Nitriliruptor*. Only the biofilm and salt crust contained a measurable population of Chlorobiota, the genus *Chlorobium* (Iino et al., 2010).

**FIGURE 4:**
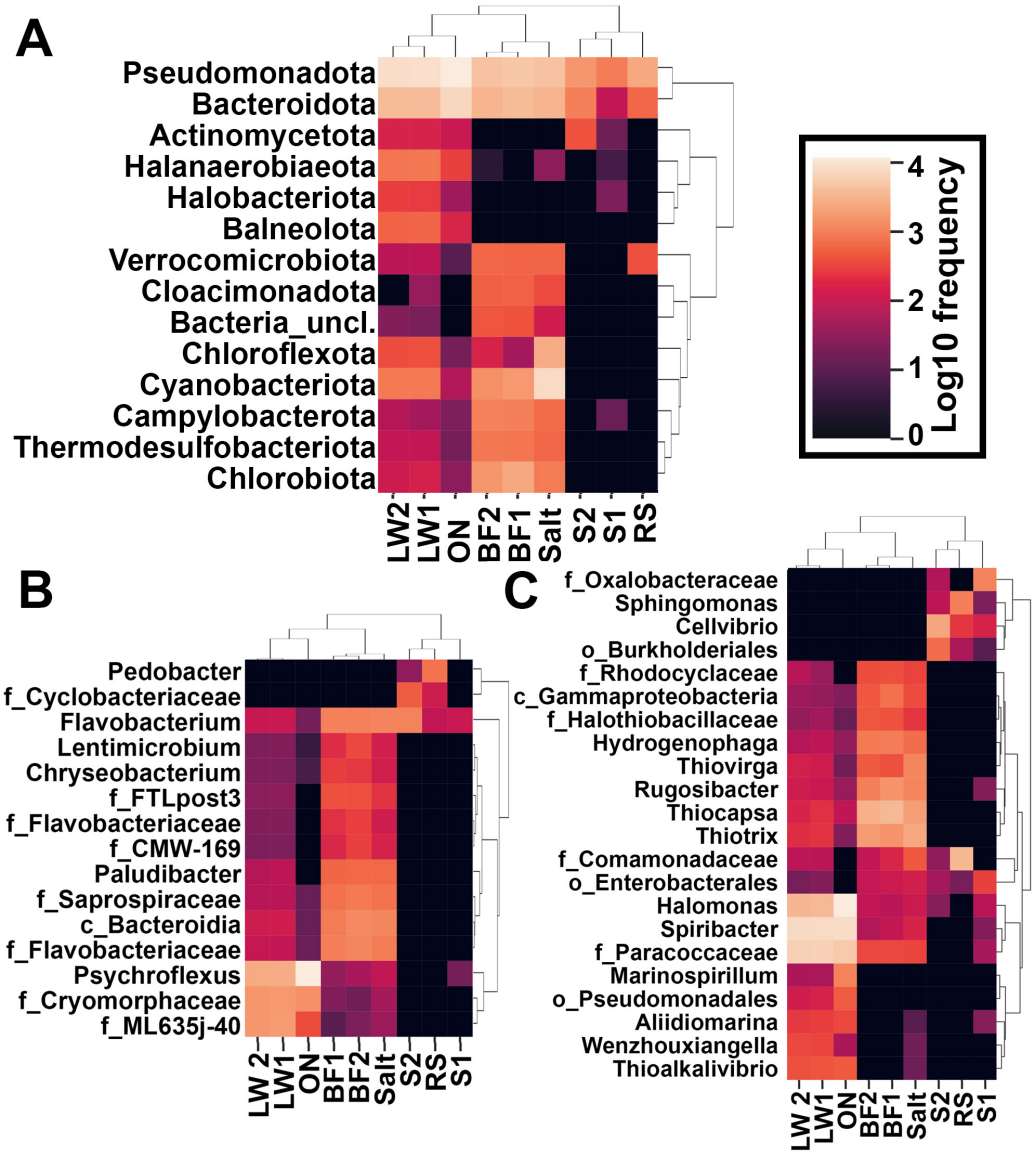
Heatmaps, filtered to remove frequencies <500. **(A)** Most common phyla. **(B)** Genera within the phylum Bacteroidota. **(C)** Genera within the phylum Pseudomonadota. f_ indicates family not further classified, c_ indicates class not further classified, and o_ indicates order not further classified.

Rarer taxa were largely unique to each site, as can be appreciated from Figure 3 as well as the heatmaps shown in Figure 4. Heatmaps at the phylum level are shown in Figure 4A. Bacteroidota and Pseudomonadota dominated all samples and were essentially the only phyla seen in the springs. Cyanobacteria were highly prevalent only in the salt crust. Some phyla were found almost exclusively in lake water: Balneolota, Halanaerobiaeota, and Halobacteriota (kingdom Archaea). Phyla common only in the biofilm/salt crust were Cloacimonadota and Verrocomicrobiota. Campylobacterota, Thermodesulfobacteriota, and Chlorobiota were prevalent in biofilm and salt crust but also present at low levels in lake water.

For ease of presentation at the genus level, heatmaps of Pseudomonadota and Bacteroidota were prepared separately. Figure 4B shows heatmaps of the most common genera within the Bacteroidota, where it can be appreciated that two classifications were found only in springs: *Pedobacter* and the family Cyclobacteriaceae. The remaining genera were found in both lake water and nearby biofilms and salt crust, but reduced or absent in the springs and the creek. The only genus found in all samples was *Flavobacterium*.

Figure 4C shows heatmaps of the most common Pseudomonadota. Here it can be appreciated that the lake water and springs contained many genera specific to those locations. Only a few genera were seen across all sites–specifically *Halomonas* and *Spiribacter*. *Sphingomonas* and *Cellvibrio* were found only in the springs; *Hydrogenophaga*, *Thiovirga*, *Thiocapsa*, and *Thiotrix* were absent from the springs.

#### 3.2.3 Picrust analysis

**FIGURE 5:**
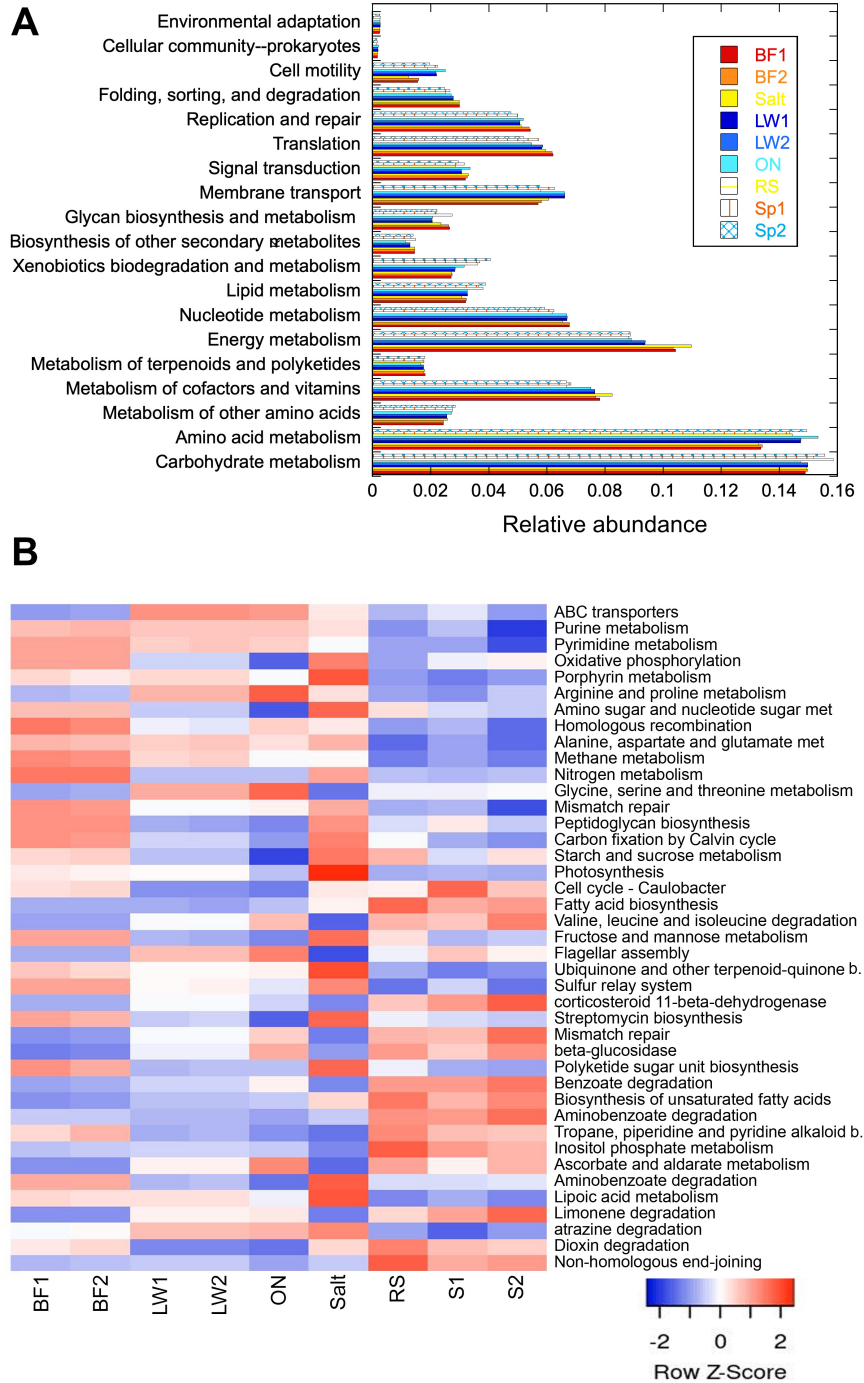
Picrust 2 metabolic pathway analysis. **(A)** General super-categories of pathway for each sample studied. **(B)** Heatmaps of specific pathways identified as significantly different between lake and biofilm or lake and springs (*p* < 0.005) by STAMP. The pathways are in order of decreasing relative abundance in the samples. When abbreviated, “b.” stands for “biosynthesis,” and “met.” indicates “metabolism.”

Picrust2 was used to predict metabolic pathways from the 16S results. NSTI scores were <2.0 for over 99% of sequences, and average NSTI scores were <0.2 for all sites, indicating good accuracy and reliability of metagenome prediction (Douglas et al., 2020) (scores given in Supplementary Table 7). The relative abundance of general classes of pathways in each of the samples is shown in Figure 5A. While the overall abundances were comparable among all samples, some clear differences were seen between the biofilm/crust samples and the liquid (lake, spring, creek) samples. Cell motility, membrane transport, and amino acid metabolism were higher in the liquid samples, while energy metabolism (which includes photosynthesis) was higher in the biofilm/salt crust. Interestingly, an increase in cell motility was seen in the lake water sample left overnight, which is consistent with imaging (not shown).

Many of the categories in Figure 5A, such as carbohydrate metabolism, are general supercategories and must be explored at deeper sub-levels to evaluate differences. Although absolute abundances using Picrust2 are expected to be different from those seen with shotgun metagenomics (Toole et al., 2021), qualitative differences may be appreciated using this method. Pathways differing between lake and springs or lake and biofilm to a statistical significance of *p* < 0.005 as calculated by STAMP are shown in an unclustered heatmap in Figure 5B. Pathways associated with nitrogen and methane metabolism were greater in biofilm and lake than in springs. Pathways associated with photosynthesis were greatly enhanced in the salt crust relative to all other samples. Several pathways related to xenobiotic degradation differed among the sites: dioxin, limonene, and benzoate pathways were enhanced in springs relative to lake, atrazine pathways higher in the lake, and aminobenzoate pathways in the biofilm and salt crust.

**FIGURE 6:**
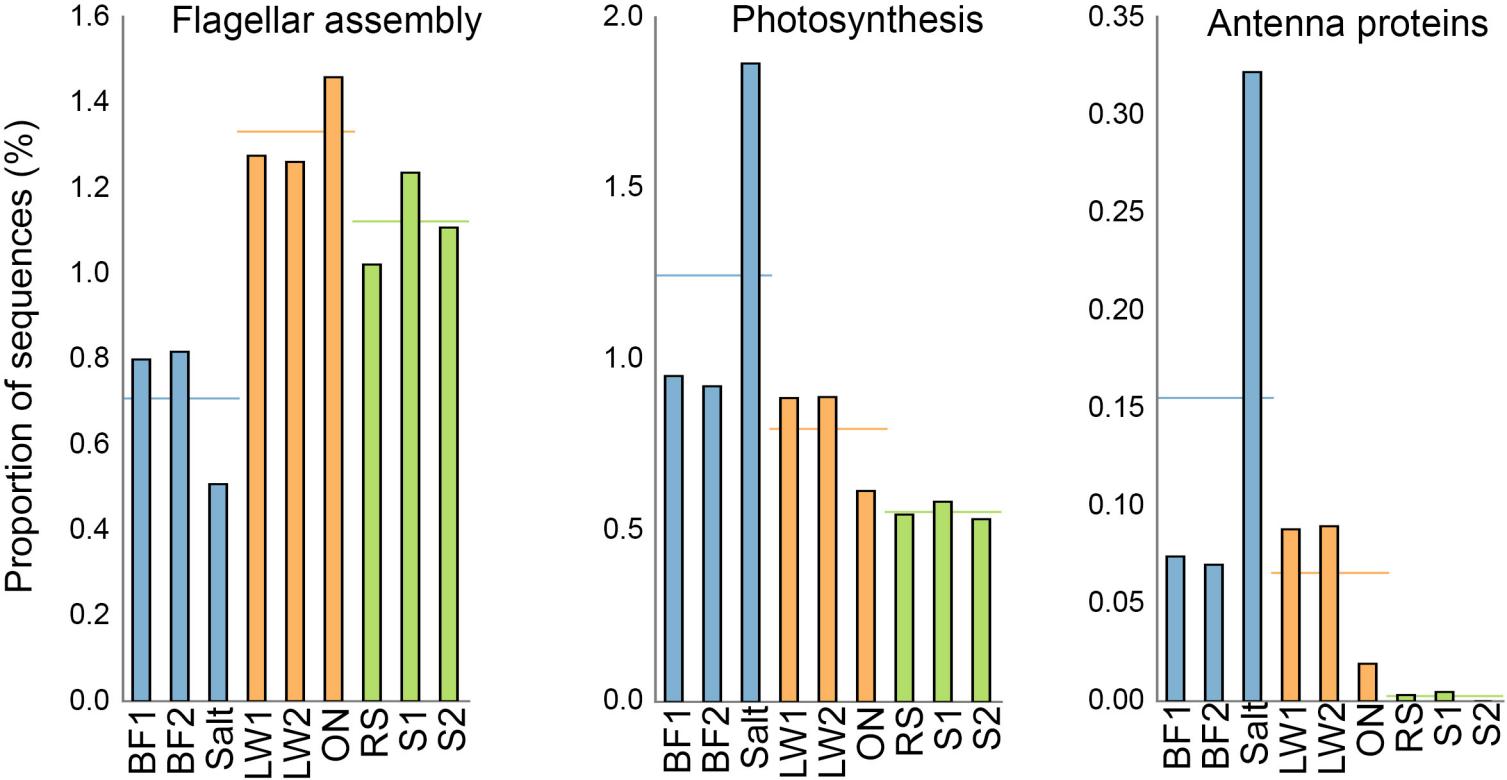
Selected pathways showing substantial differences between biofilm (BF1, BF2) and salt crust (Salt), and/or between fresh lake water (LW1, LW2) and overnight incubated lake water (ON).

For the majority of pathways, the fresh lake water samples were similar to the overnight-incubated lake water, and the biofilm was comparable to the salt crust. However, there were a few key exceptions to this, with those of particular importance shown in Figure 6. Genes for flagellar assembly were notably low in the salt crust sample, and notably high in the overnight-incubated lake water. This is consistent with the motility observations seen in Figure 5A. Genes for photosynthesis and antenna proteins were notably increased in the salt crust, consistent with the high prevalence of Cyanobacteria in this sample.

#### 3.2.4 Source tracking

**FIGURE 7:**
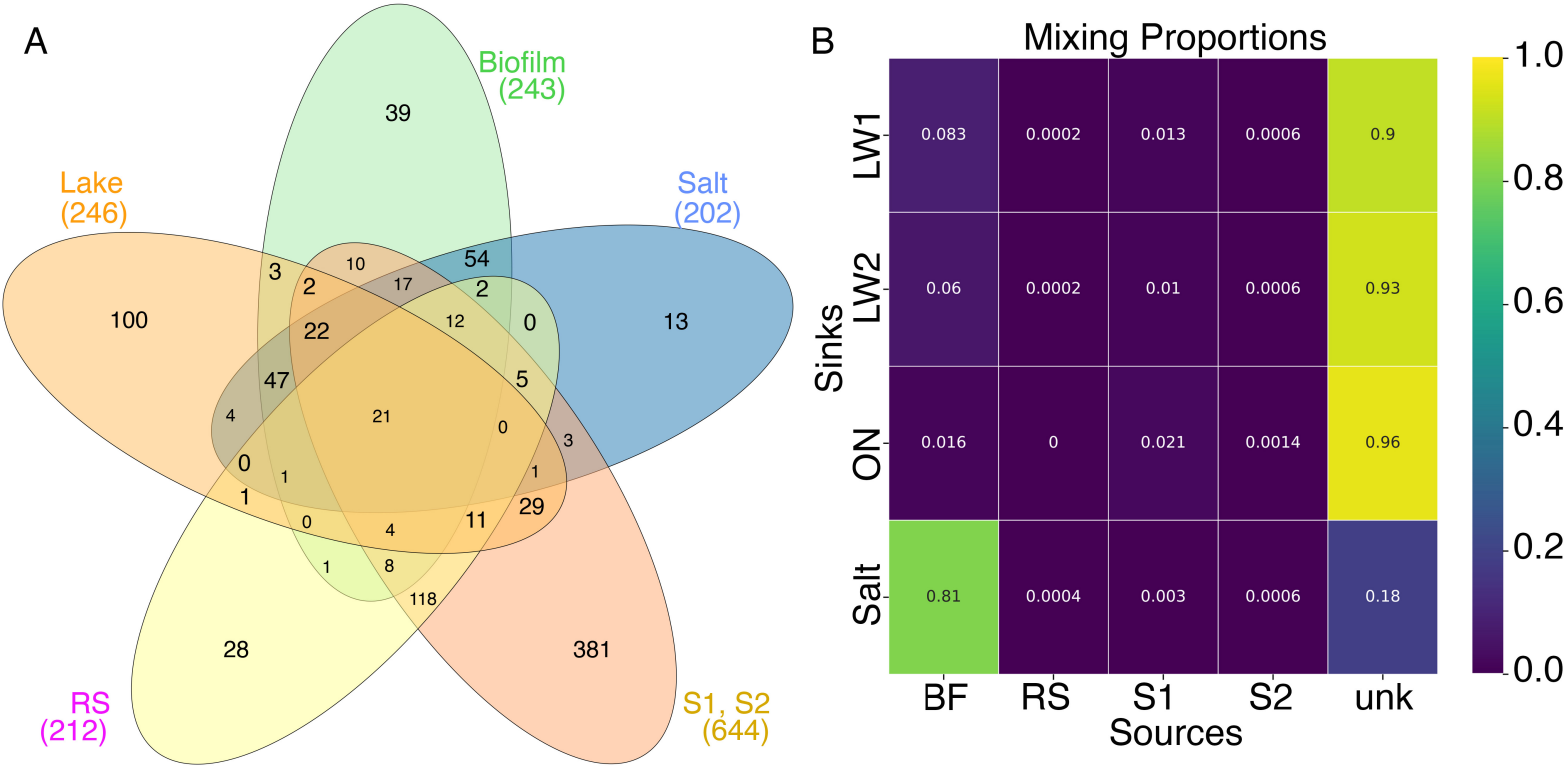
Contribution of springs to the lake. **(A)** Venn diagram of genera found in the Corral Springs biofilm, salt crust, Springs 1 and 2 (S1, S2 combined), Roberts Ranch/Wyman Creek (RS), and the lake water. **(B)** Mixing proportions calculated using SourceTracker using the lake water samples (LW1, LW2, and overnight incubated [ON]) and the salt crust as sinks, and the biofilm, RS, S1 and S2 as sources. “Unk” indicates “unknown source.” The Gibbs sampler was run for 10,000 iterations.

Venn diagrams of prokaryotic sequences showed that a large number of 16S amplicons were unique to the lake, biofilm, and springs, including 100 identified in the lake water out of 246 total, and 381 in the springs out of 644 total (Figure 7A). Bayesian source tracking analysis using the lake water and salt crust as sinks and the two springs, Corral Springs biofilm, and Robert’s Ranch/Wyman Creek as sources suggested a strong contribution of the Corral Springs biofilm sample to the salt crust, as would be expected by their proximity. The contribution of Corral Springs to the lake water was much less, and of the springs, only Spring 1 contributed to the lake water at a level >1%. Over 90% of the lake water OTUs came from unknown sources (Figure 7B). Varying rarefaction values did not substantially change the results. Analysis using an alternative algorithm, non-negative matrix factorization yielded similar results (not shown).

### 3.3 Fungi (ITS)

A high number of reads was seen in the blank sample, probably due to the dust prevalent in the laboratory. The genera occurring most frequently in the blank were removed from analysis (Supplementary Table 7).

The blank contained only 6% unclassified sequences, and >95% of the classified samples were Ascomycota and Basidiomycota. In the lake and biofilm samples, approximately 50% were only classified to the kingdom level. These fungi may be undescribed or simply not represented in the most recent (2024) UNITE database.

**FIGURE 8:**
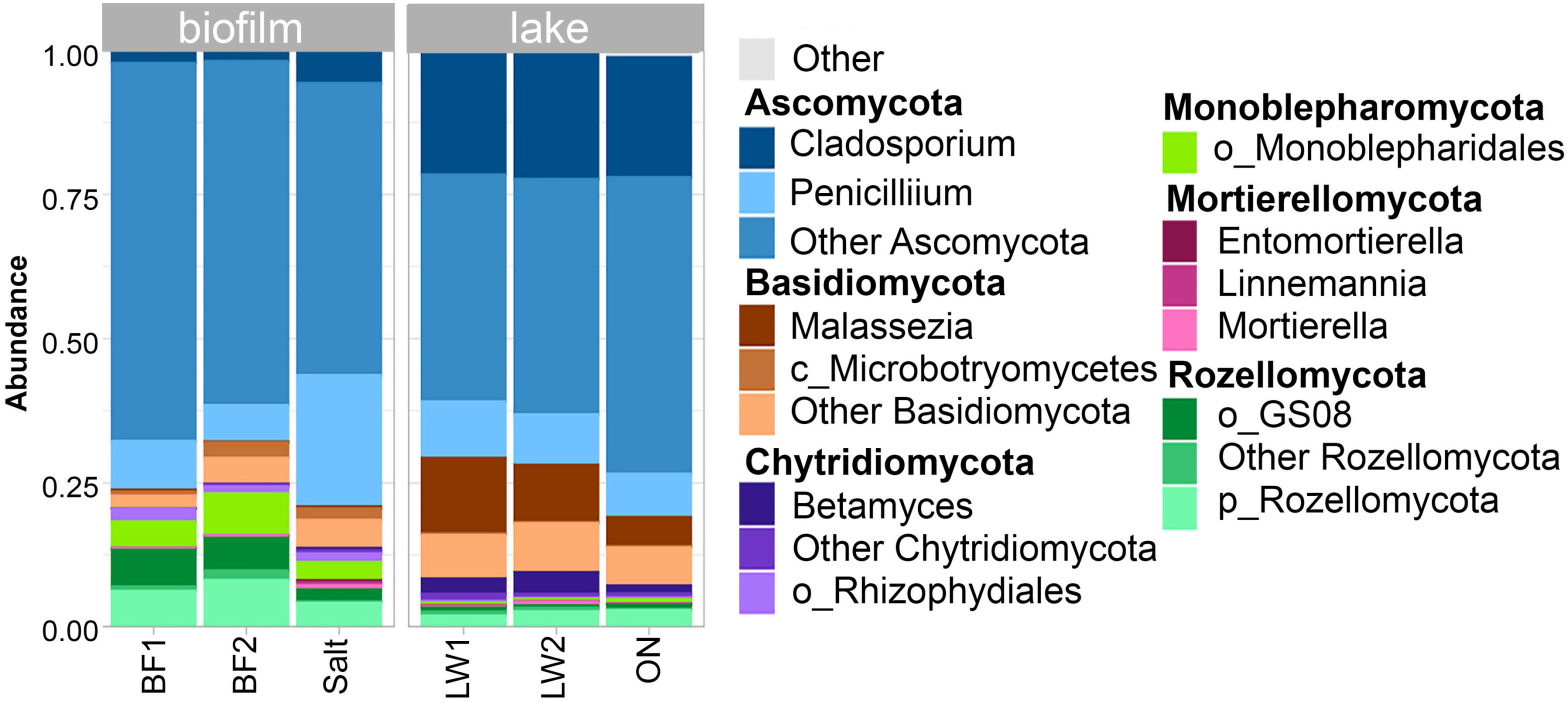
Top 2 genera of the top 6 phyla of fungi seen in the biofilm/salt crust and lake water.

When unclassified sequences and sequences more common in the blank were filtered out, the presence of representatives of 6 phyla were identified in the lake and biofilm/salt crust (Figure 8). In the lake, the most predominant single genus in the Ascomycota was *Cladosporium*, and the most common of the Basidiomycota was *Malassezia*. Rozellomycota was present at a similar level, none classified past the order level. A small signal from Mortierellomycota was also seen, all classified to the genus level as *Mortierella*, *Entomortierella*, and *Linnemannia*.

In the biofilm/salt crust samples, the Chytridomycota were largely unclassified beyond the order level. Rozellomycota were abundant, and a measurable signal was seen from Monoblepharomycota, classified only to order level. Monoblepharomycota are a sister classification to Chytridomycota. The class Microbotryomycetes in these samples includes *Rhodotorula*, and plating samples of the biofilm on peptone plates resulted in almost exclusively red colonies consistent with this genus (not shown).

**FIGURE 9:**
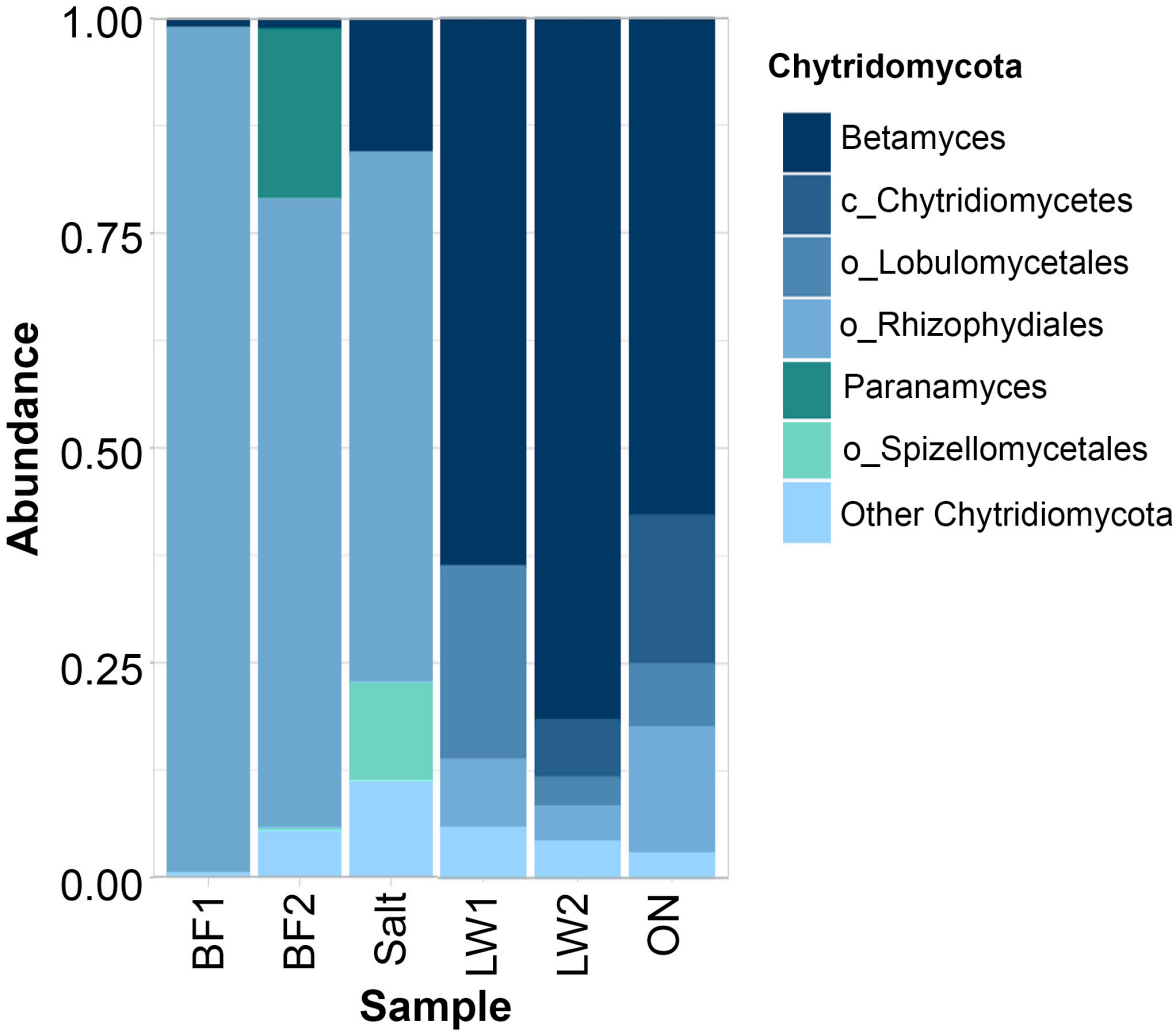
Top 6 genera (or highest other classification) of chytrid fungi in the lake and biofilm samples.

A closer look at Chytridomycota showed predominantly *Betamyces* in the lake water. The only other abundant chytrid classified to genus level was *Paranamyces* in the biofilm. Classified to order level were Lobulomycetales, Rhizophydiales, and Spizellomycetales (Figure 9).

### 3.4 Eukaryotes (18S)

**FIGURE 10:**
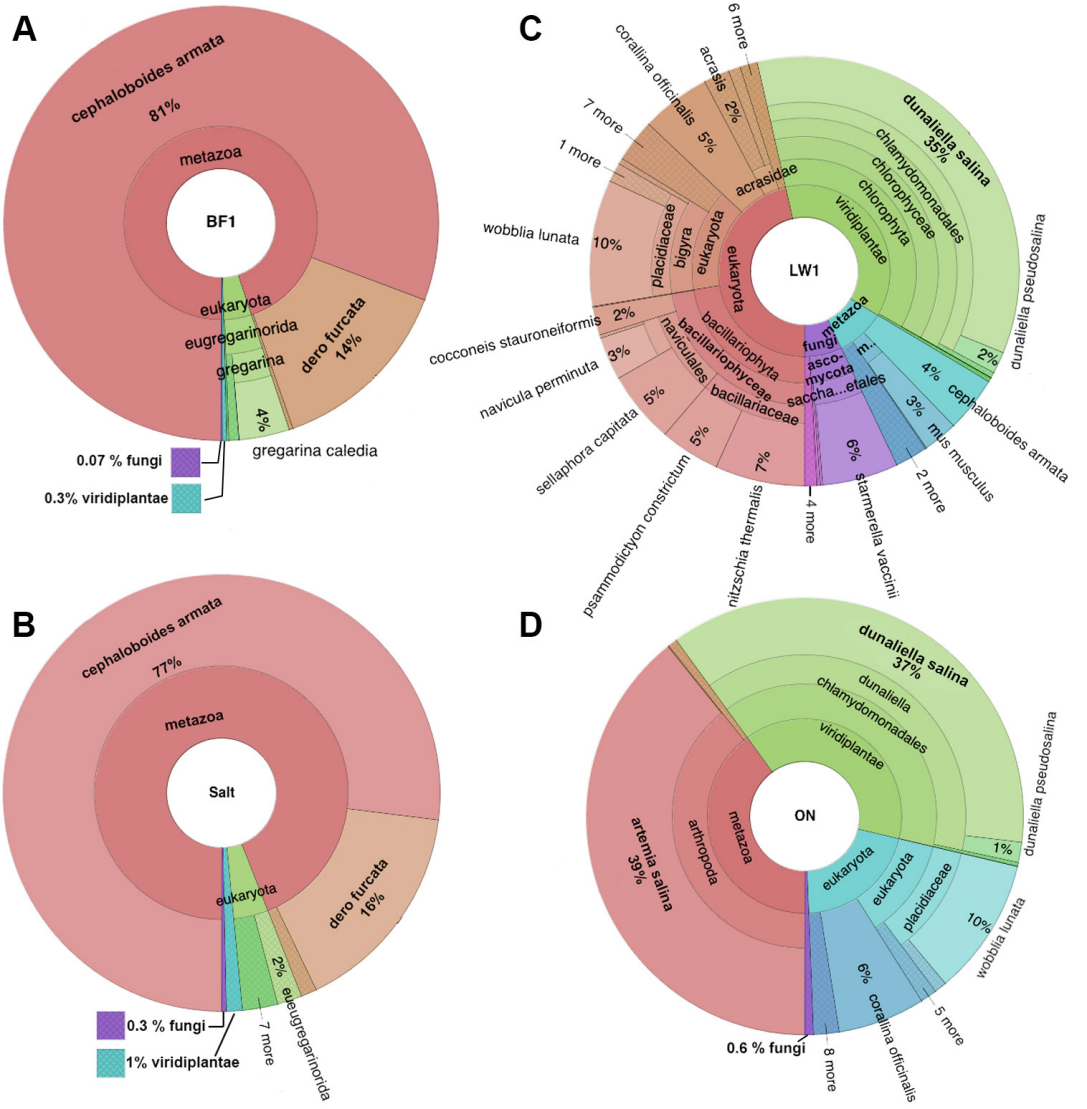
Krona plots at kingdom to species level showing results of 18S sequencing for **(A)** biofilm, **(B)** salt crust, **(C)** lake water and **(D)** overnight incubated lake water. In panel **(C)**, “saccha…etales” refers to Saccharomycetales, and m… to Mammalia.

The diversity of eukaryotes in the biofilm and salt crust was low. About 80% of the signal in these samples was classified as *Cephaloboides armata*. The second largest signal resulted from *Dero furcata*, an annelid worm. <1% of the biofilm and <2% of the salt crust sample consisted of plants and fungi (Figures 10A, B).

The lake water was richer in eukaryotic species, mostly unicellular flagellates and diatoms. The two most abundant species were *Dunaliella salina* and *Wobblia lunata*. Also present were diatoms such as *Nitzschia*, *Sellaphora*, and *Psammodictyon* (Figure 10C).

In the lake water incubated overnight, 39% of the signal came from *Artemia salina*, or brine shrimp, a species that was not apparent in the top classifications from the freshly collected water (Figure 10D).

### 3.5 Imaging

**FIGURE 11:**
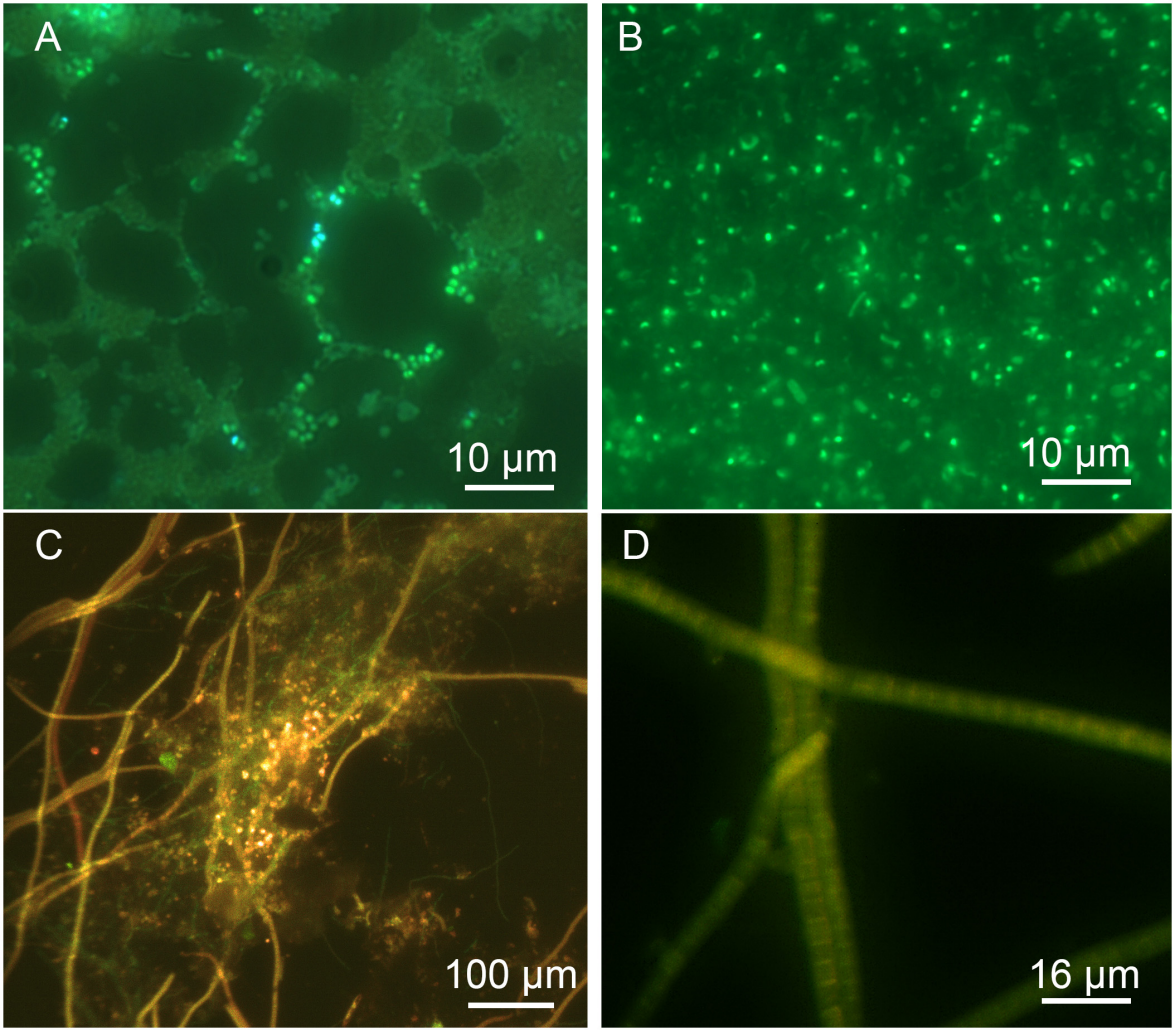
Fluorescence microscopy of spring, lake, and biofilm. **(A)** Filter-concentrated spring water stained with SYTO9. **(B)** Filter-concentrated lake water stained with SYTO9. **(C)** Biofilm stained with acridine orange. Green indicates DNA staining, red is chlorophyll autofluorescence. **(D)** Higher power image of segmented cells from biofilm.

In the lake water, especially after overnight incubation, brine shrimp were visible with a dissecting microscope or by eye, and continued to grow over several days in both size and number (not shown). Filter concentration of lake, spring, and creek water was required to obtain a countable number of bacteria in each high power field. The spring and creek water contained numerous small (<1 μm) cells that stained variably with SYTO9 (Figure 11A). The lake water showed cells of different morphologies and strong SYTO9 staining; the elongated cells were motile before drying (not shown) (Figure 11B). In the biofilm, large, segmented cells with red chlorophyll autofluorescence were abundant, along with other cells of varying morphologies that stained with acridine orange (Figures 11C, D).

## 4 Discussion

### 4.1 Overview

This paper presents the first comprehensive amplicon community composition and diversity analysis of prokaryotes, eukaryotes, and fungi of Deep Springs Lake, a soda lake in the Inyo Valley, California that has been the subject of extensive mineralogical and hydrological analysis. We identified the predominant prokaryotes and eukaryotes in the lake water and surrounding environments, and also explored the prokaryotic communities of remote feeder springs higher in the mountains. Bayesian source-sink mixing analysis suggested that the majority of organisms found in the lake did not originate from the remote springs. This is not surprising, as the lake’s extreme conditions likely drive selection and evolution of specific microbiota. Many sources other than groundwater also contribute to the lake, including runoff from rainfall/snowmelt, sediments, and contamination by birds, cattle, and human activity, as is seen in Mono Lake (Humayoun et al., 2003). This suggested that further investigation of prokaryotes, fungi, and flagellates in the lake would reveal new species with specific adaptations to high pH, highly variable salinity, and periodic drying. The contribution of the identified organisms to the nutrient cycles in the respective sites are discussed below.

### 4.2 Feeder springs

As could be expected from their near-neutral pH and low salt concentrations, the remote springs and Wyman Creek largely contained mesophilic aerobes, specifically Bacteroidota of the *Cytophaga*-*Flavobacterium* cluster, a common grouping in marine and freshwater environments (Kirchman, 2002). *Pedobacter*, a groundwater organism associated with pollution and “superbug” level resistance to antibiotics (Viana et al., 2018; Sha et al., 2023), was also found in the springs and creek. Pseudomonadota found only in the springs included the aerobic saprophyte *Cellvibrio* (DeBoy et al., 2008) as well as a collection of many other genera not shared with the lake. The spring samples showed enhanced expression of degradation pathways for several xenobiotics (dioxin, limonene, and benzoate) relative to the lake and biofilm samples, consistent with the aerobic/anaerobic balance in groundwater needed for these pathways (Carmona et al., 2009; Valderrama et al., 2012; Nhung et al., 2022). The overall picture of these springs was of neutral pH groundwater.

### 4.3 Lake water

In contrast to the springs, the organisms in the lake water showed a completely different community composition, one that has much in common with that of soda lakes worldwide (Zorz et al., 2019). Soda lake communities reflect nutrient cycling under the dual extremes of high pH and high salt concentration, where phosphate is in excess but nitrogen is a limiting nutrient (Sorokin et al., 2014, 2015a). As is commonly seen in hypersaline lakes, the flagellated unicellular green alga *Dunaliella salina* was an abundant primary producer in Deep Springs Lake, with photosynthetic prokaryotes nearly absent. The importance of this organism to primary production can be overlooked in studies where only 16S sequencing is performed (Oren, 2014). *Dunaliella* is the food of the brine shrimp *Artemia salina*, also found by 18S sequencing in the lake water and visible by eye in the samples. *Artemia* are found at a wide range of salinities and temperatures in hypersaline lakes; a unique species, *Artemia monica*, inhabits Mono Lake and exhibits two generations per year (spring and summer) with overwintering in cyst form (Drinkwater and Crowe, 1991).

Carbon degradation of the primary production is performed by aerobic and anaerobic heterotrophs, some of which can degrade polymers (primary heterotrophs) and some of which degrade the resulting monomers (secondary heterotrophs) (Sorokin et al., 2014). *ML635J-40 aquatic group* was highly abundant in the lake water, which is a heterotroph from the *Cytophaga*-*Flavobacterium*-Bacteroidetes group that is commonly found in soda lakes and carries out anaerobic biomass degradation. The abundant *Spiribacter* is a highly adaptable chemoorganoheterotroph that can use light energy and whose growth is enhanced by sulfide (Xue et al., 2021). It has been suggested as a model organism for studying adaptations to high salt and pH conditions (León et al., 2018). Other heterotrophs shared with many soda lakes included *Nitriliruptor*, which metabolizes nitriles, and *Wenzhouxiangella* (Sorokin et al., 2020).

The sulfur cycle in soda lakes has been well studied (Vavourakis et al., 2019; Sorokin et al., 2011a,b, c, 2019; Poser et al., 2013). Sulfur oxidizing bacteria were highly abundant in Deep Springs Lake, sharing genera with lakes in Europe, Asia, and elsewhere in North America. The sulfur cycle in soda lakes possesses some features different from that at lower pH; one key reason is that at high pH, sulfide is ionized to HS^–^, which is less toxic than H_2_S. The abundance of sulfur-oxidizing bacteria in soda lakes has been appreciated for over 20 years, and Deep Springs Lake is consistent with other soda lakes in its abundance of sulfur-oxidizing chemolithotrophs (*Thioalkalivibrio*) and sulfur-oxidizing heterotrophs (*Halomonas*). *Thioalkalivibrio* is the most well-studied sulfur-oxidizing bacterium from soda lakes, with over 100 genomes sequenced from diverse sites worldwide, including at least 3 lakes in California (Berben et al., 2019; Ahn et al., 2017, 2019; Berben et al., 2015a,b; Sorokin and Kuenen, 2005b). Sulfur reducers identified in the lake water were *Desulfonatronobacter* and *Desulfonatronospira*, genera of lithotrophic obligate anaerobes (Sorokin et al., 2015b). They were found in low abundances from our surface samples, but might be more prevalent deeper in the water.

In hypersaline environments, the nitrogen cycle is strongly dependent upon Haloarchaea (Bonete et al., 2008). Denitrification is the process where nitrate (NO_3_), nitrite (NO_2_), nitric oxide (NO) and nitrous oxide (N_2_O) are reduced successively to gaseous nitrogen (N_2_). The family Haloferaceae, which accounted for >5% of the sequences in the lake water, consists of 74.1% species that are partial denitrifiers and 14.8% that are complete denitrifiers (Miralles-Robledillo et al., 2021).

Picrust2 pathway prediction was consistent with community composition and observation. Although Picrust2 prediction of metabolic pathways can be inaccurate relative to shotgun metagenomics, it has shown substantial success in environmental samples, including hypersaline and contaminated environments; its database has been recently updated (Li et al., 2022; Toole et al., 2021; Vera et al., 2022; Wright and Langille, 2025), and the low NSTI scores we observed suggest that the results were reliable. One notable metabolic pathway predicated to be high in the lake relative to the other sites was ABC transporters, associated with nutrient uptake from oligotrophic environments and resistance to salt, pH extremes, and toxins in both bacteria and Archaea (Albers et al., 2004; Young and Holland, 1999; Giuliani et al., 2011). Other pathways predicted to be enhanced in the lake water were purine and pyrimidine metabolism, and the pathway alanine, aspartate, and glutamate metabolism; these are all indicative of nitrogen being a limiting nutrient. The exact metabolisms used by these organisms will only be known once they are sequenced to the species or even strain level. Some genera, such as *Thioalkalivibrio*, show highly diverse metabolisms at the species level (Sorokin and Kuenen, 2005a).

The question of how quickly samples need to be preserved is important in environmental sequencing studies, where dry ice is often unavailable and samples must be carried from remote sites on foot. Accordingly, we were interested in the effects of storage, and observed that the microbial communities showed striking changes in a sample of lake water left in a collection vessel for 24 h before DNA extraction. Most notable were a complete loss of Archaea in the incubated sample and the proliferation of *Artemia salina*. It is known from hydrological studies of Deep Springs Lake that pH and alkalinity values change rapidly after collection (Jones, 1965), allowing for the proliferation of organisms not adapted to high pH. These results underscore the importance of immediate freezing or DNA extraction from this site, likely generalizable to other soda lakes. This is in contrast to soil or human samples, where studies have reported that several days of storage at temperatures from −20 °C to +20 °C does not substantially affect community composition (Lauber et al., 2010; Rubin et al., 2013).

### 4.4 Biofilm and salt crust

Bacterial communities in the dried salt crust immediately adjacent to the lake were much more compositionally similar to the upstream biofilm-containing channels than to those in the lake water itself. These communities resembled those found in other saline microbial mats (Hentschke et al., 2025), distinct from the soda lake environment in several respects. Corral Creek biofilm and salt crust samples were the only sites showing abundant populations of photosynthetic prokaryotes, especially Cyanobacteria, which help form the structure of mats (Nguyen et al., 2022). *Thiocapsa*, one of the most studied lineages of photosynthetic sulfur bacteria (Gitelson et al., 1999; Wu et al., 2021), made up ∼8% of sequences in the biofilm and slightly less in the salt crust.

The biofilm/salt crust also contained genera of sulfur oxidizers. The “sulfur relay system” (KEGG ko04122) was identified as a pathway enhanced in these samples by Picrust2, consisting of sulfur carrier proteins and enzymes involved in sulfur transfer. *Chlorobium*, of the phylum Chlorobiota, was abundant. This is a photolithotrophic sulfur oxidizer that lives in strictly anaerobic conditions below the surface of a body of water, commonly the anaerobic zone of a eutrophic lake (Gorlenko et al., 2023; Grouzdev et al., 2022). Present at a lower level were *Thiotrix* and *Sulfurovum*, which form filaments in cold and hot sulfur springs (Reigstad et al., 2011; Nosalova et al., 2023), and *Thiovirga* (Ito et al., 2005).

A number of genera of sulfur reducing bacteria (SRB) were identified in the biofilm/salt crust, largely distinct from those seen in the lake water. These included the abundant *Trichlorobacter* and the less common *Desulfomicrobium* and *Desulfocapsa* (Finster et al., 2013).

Also present only in these samples was the genus *Rugosibacter*, which are organisms capable of degrading polycyclic aromatic hydrocarbons, recently reclassified from the family Thodocyclaceae to Sterolibacteriaceae (Corteselli et al., 2017; Kojima et al., 2022). *Hydrogenophaga*, a hydrogen oxidizer, was also present; hydrogen is generated from cyanobacteria in microbial mats and consumed by hydrogen oxidizers such as *Hydrogenophaga* and SRB, which may compete or occupy distinct niches in processes of hydrogen cycling that are just beginning to be understood (Kong et al., 2025; Nielsen et al., 2015; Maegaard et al., 2017).

Metabolic pathways predicted to be enhanced in biofilm/salt crust included those associated with autotrophs, including oxidative phosphorylation, porphyrin metabolism, carbon fixation by the Calvin cycle, and photosynthesis. Nitrogen metabolism was also significantly enhanced, but the KEGG classification (k00910) for nitrogen metabolism includes all processes in the nitrogen cycle (nitrogen fixation, nitrification, denitrification) and so relates to many diverse taxa without distinction. *Oscillatoria* is a known nitrogen fixer; cyanobacterial mats obtain both fixed carbon and nitrogen from the cyanobacteria. *Trichlorobacter* can also reduce nitrate to ammonium (Sorokin et al., 2023; Tabares et al., 2025).

The unique endemic black toad, *Anaxyrus Exsul*, lives in Corral Springs and other springs near the lake, but does not inhabit the hypersaline, high pH environment of the lake itself (Wang, 2009). Therefore, examining the community composition of the biofilm samples may shed some light into the microbiota of these endangered animals. For the Sierra Nevada yellow-legged frog (*Rana sierrae*), it has been found that the most common bacterial order on the skin microbiome is Burkholderiales, which is also the only order negatively correlated with alpha-diversity (Ellison et al., 2021). In our samples, several families of Burkholderiales were identified in the biofilm: Comamondaceae (genus *Hydrogenophaga* and genera not otherwise classified) and Rhodocyclaceae, genus unclassified, for a total of 10% of reads for the order. Direct sampling of the toad microbiome by skin swab will allow for comparison of these taxa with those in the water samples.

Table 1 gives examples of metabolic functions and representative taxa found in the lake water and biofilm/salt crust, with the caveat that many of these organisms are mixotrophs and so may not be strictly assigned to a single category.

**TABLE 1:** Representative taxa taking part in carbon, nitrogen, and sulfur cycling in the Deep Springs Lake area.

	Lake water	Biofilm/salt crust
Photoautotrophs	*Dunaliella* (eukaryote)	Cyanobacteria (*Oscillatoria*, *Kamptonema*, *Planktothricoides*) *Thiocapsa*
Sulfur oxidizers	*Thioalkalivibrio* *Halomonas*	*Chlorobium* *Thiotrix*
Sulfur reducers	*Desulfonatronobacter* *Halalkaliarchaeum*	*Desulfocapsa* *Trichlorobacter*
Biomass degradation	*Spiribacter* *ML635J-40 aquatic group*	*Rugosibacter* *Paludibacter* *Hydrogenophaga*
Nitrogen cycle	Haloferaceae (Archaea) (denitrification)	*Oscillatoria* (nitrogen fixation) *Trichlorobacter* (nitrate reduction)

### 4.5 Fungi

In contrast to bacteria and eukaryotes, fungi have not been well studied in soda lakes. Evaluation of fungal communities by ITS sequencing in Deep Springs Lake was approximate, given the high number of sequences not classified past the kingdom level. However, the results we found were environmentally relevant. Chytrid fungi of the genus *Batrachochytrium* have been associated with amphibian die-offs (Zipkin and DiRenzo, 2022). In our samples of biofilm, salt crust, and lake water, the phylum Chytridomycota yielded a substantial signal, but most could be classified into the genus *Betamyces*, first reported from Argentina pollen baits and later seen in take sediments from Antarctica and soils from both Antarctica and Costa Rica (Gonçalves et al., 2022; Krings and Harper, 2018; Letcher et al., 2012) and from Arctic and Antarctic lakes (Marchetta et al., 2023). No sign of amphibian pathogens was found here.

Other fungi of environmental interest were also seen in the samples. The most predominant single genus in the Ascomycota was *Cladosporium*, and the most common of the Basidiomycota was *Malassezia*, a lipophilic yeast associated with skin infections in humans and animals (Velegraki et al., 2015; Hobi et al., 2022). The ubiquity and diversity of *Malassezia* in marine environments has only recently come to attention (Steinbach et al., 2023). Improved databases of fungi from extreme environments and study of individual isolates of culturable species could assist in improving the ability to classify fungi from these sites.

## 5 Conclusion

This study represents the first investigation into the microbial, fungal, and eukaryotic diversity in Deep Springs Lake, a previously uncharacterized soda lake in the Inyo Valley, California. The results show characteristic taxa shared with high-pH, hypersaline systems worldwide. The microbiota of the springs feeding the lake have little overlap with those of the lake water itself, suggesting specific adaptations to this extreme environment that may include evolution of unique species of interest to biotechnology and astrobiology.

Other avenues of future investigation include following the populations of selected organisms or entire biomes seasonally, which could be expected to show large variations in populations of *Artemia*, photosynthetic organisms, and bacteria that are preyed upon by *Artemia*, as is seen in Mono Lake (Melack et al., 2017). Culturing and whole genome sequencing of selected organisms will show whether the organisms in the lake are unique species and would help evaluate the fungi further. Identification of unique species would be interesting it its own right, but also provide evolutionary context for comparison with other soda lakes, both near and far.

## Data Availability

The datasets presented in this study can be found in online repositories. The names of the repository/repositories and accession number(s) can be found below: https://www.ncbi.nlm.nih.gov/bioproject/1292893.

## References

[B1] Abarenkov K. Nilsson R. H. Larsson K. H. Taylor A. F. S. May T. W. Frøslev T. G. (2024). The UNITE database for molecular identification and taxonomic communication of fungi and other eukaryotes: Sequences, taxa and classifications reconsidered. *Nucleic Acids Res.* 52 D791–D797. 10.1093/nar/gkad1039 37953409 PMC10767974

[B2] Ahn A. C. Cavalca L. Colombo M. Schuurmans J. M. Sorokin D. Y. Muyzer G. (2019). Transcriptomic analysis of two thioalkalivibrio species under arsenite stress revealed a potential candidate gene for an alternative arsenite oxidation pathway. *Front. Microbiol.* 10:1514. 10.3389/fmicb.2019.01514 31333619 PMC6620896

[B3] Ahn A. C. Meier-Kolthoff J. P. Overmars L. Richter M. Woyke T. Sorokin D. Y. (2017). Genomic diversity within the haloalkaliphilic genus Thioalkalivibrio. *PLoS One* 12:e0173517. 10.1371/journal.pone.0173517 28282461 PMC5345834

[B4] Albers S. V. Koning S. M. Konings W. N. Driessen A. J. (2004). Insights into ABC transport in archaea. *J. Bioenerg. Biomembr.* 36 5–15. 10.1023/b:jobb.0000019593.84933.e6 15168605

[B5] Berben T. Overmars L. Sorokin D. Y. Muyzer G. (2019). Diversity and distribution of sulfur oxidation-related genes in thioalkalivibrio, a genus of chemolithoautotrophic and haloalkaliphilic sulfur-oxidizing bacteria. *Front. Microbiol.* 10:160. 10.3389/fmicb.2019.00160 30837958 PMC6382920

[B6] Berben T. Sorokin D. Y. Ivanova N. Pati A. Kyrpides N. Goodwin L. A. (2015a). Complete genome sequence of *Thioalkalivibrio paradoxus* type strain ARh 1(T), an obligately chemolithoautotrophic haloalkaliphilic sulfur-oxidizing bacterium isolated from a Kenyan soda lake. *Stand. Genomic Sci.* 10:105. 10.1186/s40793-015-0097-7 26594306 PMC4653848

[B7] Berben T. Sorokin D. Y. Ivanova N. Pati A. Kyrpides N. Goodwin L. A. (2015b). Partial genome sequence of the haloalkaliphilic soda lake bacterium *Thioalkalivibrio thiocyanoxidans* ARh 2(T). *Stand. Genomic Sci.* 10:85. 10.1186/s40793-015-0078-x 26512310 PMC4624188

[B8] Bonete M. J. Martínez-Espinosa R. M. Pire C. Zafrilla B. Richardson D. J. (2008). Nitrogen metabolism in haloarchaea. *Saline Syst.* 4:9. 10.1186/1746-1448-4-9 18593475 PMC2483277

[B9] Brown C. M. Mathai P. P. Loesekann T. Staley C. Sadowsky M. J. (2019). Influence of library composition on sourcetracker predictions for community-based microbial source tracking. *Environ. Sci. Technol.* 53 60–68. 10.1021/acs.est.8b04707 30475593

[B10] Callahan B. J. McMurdie P. J. Rosen M. J. Han A. W. Johnson A. J. Holmes S. P. (2016). DADA2: High-resolution sample inference from Illumina amplicon data. *Nat. Methods* 13 581–583. 10.1038/nmeth.3869 27214047 PMC4927377

[B11] Carmona M. Zamarro M. T. Blázquez B. Durante-Rodríguez G. Juárez J. F. Valderrama J. A. (2009). Anaerobic catabolism of aromatic compounds: A genetic and genomic view. *Microbiol. Mol. Biol. Rev.* 73 71–133. 10.1128/MMBR.00021-08 19258534 PMC2650882

[B12] Carrozzo F. G. De Sanctis M. C. Raponi A. Ammannito E. Castillo-Rogez J. Ehlmann B. L. (2018). Nature, formation, and distribution of carbonates on Ceres. *Sci. Adv.* 4:e1701645. 10.1126/sciadv.1701645 29546235 PMC5851657

[B13] Case N. Johnston N. Nadeau J. (2024). Fluorescence microscopy with deep UV, near UV, and visible excitation for in situ detection of microorganisms. *Astrobiology* 24 300–317. 10.1089/ast.2023.0020 38507693 PMC10979697

[B14] Choi J. Park J. S. (2020). Comparative analyses of the V4 and V9 regions of 18S rDNA for the extant eukaryotic community using the Illumina platform. *Sci. Rep.* 10:6519. 10.1038/s41598-020-63561-z 32300168 PMC7162856

[B15] Corteselli E. M. Aitken M. D. Singleton D. R. (2017). *Rugosibacter aromaticivorans* gen. nov., sp. nov., a bacterium within the family Rhodocyclaceae, isolated from contaminated soil, capable of degrading aromatic compounds. *Int. J. Syst. Evol. Microbiol.* 67 311–318. 10.1099/ijsem.0.001622 27902243 PMC5797942

[B16] DeBoy R. T. Mongodin E. F. Fouts D. E. Tailford L. E. Khouri H. Emerson J. B. (2008). Insights into plant cell wall degradation from the genome sequence of the soil bacterium Cellvibrio japonicus. *J. Bacteriol.* 190 5455–5463. 10.1128/JB.01701-07 18556790 PMC2493263

[B17] Douglas G. M. Maffei V. J. Zaneveld J. R. Yurgel S. N. Brown J. R. Taylor C. M. (2020). PICRUSt2 for prediction of metagenome functions. *Nat. Biotechnol.* 38 685–688. 10.1038/s41587-020-0548-6 32483366 PMC7365738

[B18] Drinkwater L. E. Crowe J. H. (1991). Hydration state, metabolism, and hatching of mono lake artemia cysts. *Biol. Bull.* 180 432–439. 10.2307/1542343 29304650

[B19] Edelstein A. D. Tsuchida M. A. Amodaj N. Pinkard H. Vale R. D. Stuurman N. (2014). Advanced methods of microscope control using μManager software. *J. Biol. Methods* 1:e10. 10.14440/jbm.2014.36 25606571 PMC4297649

[B20] Ellison S. Knapp R. Vredenburg V. (2021). Longitudinal patterns in the skin microbiome of wild, individually marked frogs from the Sierra Nevada. California. *ISME Commun.* 1:45. 10.1038/s43705-021-00047-7 37938625 PMC9723788

[B21] Estaki M. Jiang L. Bokulich N. A. McDonald D. González A. Kosciolek T. (2020). QIIME 2 enables comprehensive end-to-end analysis of diverse microbiome data and comparative studies with publicly available data. *Curr. Protoc. Bioinform.* 70:e100. 10.1002/cpbi.100 32343490 PMC9285460

[B22] Fadeev E. Cardozo-Mino M. G. Rapp J. Z. Bienhold C. Salter I. Salman-Carvalho V. (2021). Comparison of two 16S rRNA primers (V3-V4 and V4-V5) for studies of arctic microbial communities. *Front. Microbiol.* 12:637526. 10.3389/fmicb.2021.637526 33664723 PMC7920977

[B23] Finster K. W. Kjeldsen K. U. Kube M. Reinhardt R. Mussmann M. Amann R. (2013). Complete genome sequence of Desulfocapsa sulfexigens, a marine deltaproteobacterium specialized in disproportionating inorganic sulfur compounds. *Stand. Genomic Sci.* 8 58–68. 10.4056/sigs.3777412 23961312 PMC3739170

[B24] Getenet M. Otálora F. Emmerling F. Al-Sabbagh D. García-Ruiz J. M. (2023). Mineral precipitation and hydrochemical evolution through evaporitic processes in soda brines (East African Rift Valley). *Chem. Geol.* 616:121222. 10.1016/j.chemgeo.2022.121222 PMC8991015 35401055

[B25] Gitelson A. Stark R. Dor I. Michelson O. Yacobi Y. Z. (1999). Optical characteristics of the phototroph *Thiocapsa roseopersicina* and implications for real-time monitoring of the bacteriochlorophyll concentration. *Appl. Environ. Microbiol.* 65 3392–3397. 10.1128/AEM.65.8.3392-3397.1999 10427024 PMC91509

[B26] Giuliani S. E. Frank A. M. Corgliano D. M. Seifert C. Hauser L. Collart F. R. (2011). Environment sensing and response mediated by ABC transporters. *BMC Genom.* 12 (Suppl. 1):S8. 10.1186/1471-2164-12-S1-S8 21810210 PMC3223731

[B27] Glein C. R. Baross J. A. Waite J. H. (2015). The pH of Enceladus’ ocean. *Geochim. Cosmochimica Acta* 162 202–219. 10.1016/j.gca.2015.04.017

[B28] Gonçalves V. N. de Souza L. M. D. Lirio J. M. Coria S. H. Lopes F. A. C. Convey P. (2022). Diversity and ecology of fungal assemblages present in lake sediments at Clearwater Mesa, James Ross Island, Antarctica, assessed using metabarcoding of environmental DNA. *Fungal Biol.* 126 640–647. 10.1016/j.funbio.2022.08.002 36116896

[B29] Gorlenko V. Savvichev A. Kadnikov V. Rusanov I. Beletsky A. Zakharova E. (2023). A novel view of the diversity of anoxygenic phototrophic bacteria inhabiting the chemocline of meromictic karst lakes. *Microorganisms* 12:13. 10.3390/microorganisms12010013 38276182 PMC10820006

[B30] Grouzdev D. Gaisin V. Lunina O. Krutkina M. Krasnova E. Voronov D. (2022). Microbial communities of stratified aquatic ecosystems of Kandalaksha Bay (White Sea) shed light on the evolutionary history of green and brown morphotypes of Chlorobiota. *FEMS Microbiol. Ecol.* 98:fiac103. 10.1093/femsec/fiac103 36073352

[B31] Haas S. Sinclair K. P. Catling D. C. (2024). Biogeochemical explanations for the world’s most phosphate-rich lake, an origin-of-life analog. *Commun. Earth Environ.* 5:28. 10.1038/s43247-023-01192-8

[B32] Haines M. Khot V. Strous M. (2023). The vigor, futility, and application of microbial element cycles in alkaline soda lakes. *Elements* 19 30–36. 10.2138/gselements.19.1.30

[B33] Hake K. H. West P. T. McDonald K. Laundon D. Reyes-Rivera J. Garcia de las bayonas A. (2024). a large colonial choanoflagellate from mono lake harbors live bacteria. *mBio* 15:e0162324. 10.1128/mbio.01623-24 39140743 PMC11389367

[B34] Halko N. Martinsson P.-G. Shkolnisky Y. Tygert M. (2011). An algorithm for the principal component analysis of large data sets. *SIAM J. Sci. Comp.* 33 2580–2594. 10.1137/100804139

[B35] Hamady M. Lozupone C. Knight R. (2010). Fast UniFrac: Facilitating high-throughput phylogenetic analyses of microbial communities including analysis of pyrosequencing and PhyloChip data. *ISME J.* 4 17–27. 10.1038/ismej.2009.97 19710709 PMC2797552

[B36] Hentschke G. S. Semedo M. Ciancas J. Hoepfner C. Guzmán D. Rivera D. S. (2025). Cyanobacterial mats and their associated microbiomes in saline and freshwater lakes from the Bolivian Altiplano. *Front. Microbiol.* 16:1650455. 10.3389/fmicb.2025.1650455 40771682 PMC12325342

[B37] Hobi S. Cafarchia C. Romano V. Barrs V. R. (2022). Malassezia: Zoonotic implications, parallels and differences in colonization and disease in humans and animals. *J. Fungi* 8:708. 10.3390/jof8070708 35887463 PMC9324274

[B38] Huang Z. Cai D. Sun Y. (2024). Towards more accurate microbial source tracking via non-negative matrix factorization (NMF). *Bioinformatics* 40 (Suppl. 1), i68–i78. 10.1093/bioinformatics/btae227 38940128 PMC11256951

[B39] Humayoun S. B. Bano N. Hollibaugh J. T. (2003). Depth distribution of microbial diversity in Mono Lake, a meromictic soda lake in California. *Appl. Environ. Microbiol.* 69 1030–1042. 10.1128/AEM.69.2.1030-1042.2003 12571026 PMC143613

[B40] Iino T. Mori K. Uchino Y. Nakagawa T. Harayama S. Suzuki K. I. (2010). *Ignavibacterium album* gen. nov., sp. nov., a moderately thermophilic anaerobic bacterium isolated from microbial mats at a terrestrial hot spring and proposal of Ignavibacteria classis nov., for a novel lineage at the periphery of green sulfur bacteria. *Int. J. Syst. Evol. Microbiol.* 60(Pt 6), 1376–1382. 10.1099/ijs.0.012484-0 19671715

[B41] Ito T. Sugita K. Yumoto I. Nodasaka Y. Okabe S. (2005). *Thiovirga sulfuroxydans* gen. nov., sp. nov., a chemolithoautotrophic sulfur-oxidizing bacterium isolated from a microaerobic waste-water biofilm. *Int. J. Syst. Evol. Microbiol.* 55(Pt 3), 1059–1064. 10.1099/ijs.0.63467-0 15879233

[B42] Jani A. J. Briggs C. J. (2018). Host and aquatic environment shape the amphibian skin microbiome but effects on downstream resistance to the pathogen batrachochytrium dendrobatidis are variable. *Front. Microbiol.* 9:487. 10.3389/fmicb.2018.00487 29619014 PMC5871691

[B43] Jones B. F. (1965). *The hydrology and mineralogy of Deep Springs Lake, Inyo County, California.* Washington, DC: United States Geological Survey.

[B44] Junkins E. N. Stamps B. W. Corsetti F. A. Oremland R. S. Spear J. R. Stevenson B. S. (2019). Draft genome sequence of picocystis sp. strain ml, cultivated from mono lake, California. *Microbiol. Resour. Announc.* 8:e01353-18. 10.1128/MRA.01353-18 30701234 PMC6346183

[B45] Kanehisa M. (2002). “The KEGG Database,”. in *In Silico Simulation of Biological Processes* eds Novartis Foundation, Bock G. Goode J.A. 10.1002/0470857897.ch8

[B46] Kanzaki N. Yamashita T. Lee J. S. Shih P. Y. Ragsdale E. J. Shinya R. (2021). Tokorhabditis n. gen. (Rhabditida, Rhabditidae), a comparative nematode model for extremophilic living. *Sci. Rep.* 11:16470. 10.1038/s41598-021-95863-1 34389775 PMC8363662

[B47] Kempe S. Kazmierczak J. (2002). Biogenesis and early life on Earth and Europa: Favored by an alkaline ocean? *Astrobiology* 2 123–130. 10.1089/153110702753621394 12449860

[B48] Kirchman D. L. (2002). The ecology of Cytophaga-Flavobacteria in aquatic environments. *FEMS Microbiol. Ecol.* 39 91–100. 10.1111/j.1574-6941.2002.tb00910.x 19709188

[B49] Knights D. Kuczynski J. Charlson E. S. Zaneveld J. Mozer M. C. Collman R. G. (2011). Bayesian community-wide culture-independent microbial source tracking. *Nat. Methods* 8 761–763. 10.1038/nmeth.1650 21765408 PMC3791591

[B50] Knott J. R. Mahan S. A. Bright J. Langer L. Ramirez A. Mccarty K. (2023). Pliocene–Pleistocene hydrology and pluvial lake during marine isotope stages 5a and 4, deep springs valley, western great basin, inyo county, California. *Quaternary Res.* 115 160–178. 10.1017/qua.2023.20

[B51] Kojima H. Watanabe M. Miyata N. Fukui M. (2022). *Sulfuricystis multivorans* gen. nov., sp. nov. and *Sulfuricystis thermophila* sp. nov., facultatively autotropic sulfur-oxidizing bacteria isolated from a hot spring, and emended description of the genus Rugosibacter. *Arch. Microbiol.* 204:595. 10.1007/s00203-022-03186-0 36053377

[B52] Kong L. Feng Y. Zheng R. Wu X. Mao Y. Sun J. (2025). Interspecies hydrogen transfer between cyanobacteria and symbiotic bacteria drives nitrogen loss. *Nat. Commun.* 16:5078. 10.1038/s41467-025-60327-x 40450007 PMC12126579

[B53] Krings M. Harper C. J. (2018). Deciphering interfungal relationships in the 410million-year-old Rhynie chert: Glomoid spores under attack. *Geobios* 51 151–160. 10.1016/j.geobios.2018.02.004

[B54] Lam B. A. Walke J. B. Vredenburg V. T. Harris R. N. (2010). Proportion of individuals with anti-Batrachochytrium dendrobatidis skin bacteria is associated with population persistence in the frog Rana muscosa. *Biol. Conserv.* 143 529–531. 10.1016/j.biocon.2009.11.015

[B55] Langbein W. B. (1961). *Salinity and hydrology of closed lakes.* Washington, D.C: U.S. Government Printing Office.

[B56] Lauber C. L. Zhou N. Gordon J. I. Knight R. Fierer N. (2010). Effect of storage conditions on the assessment of bacterial community structure in soil and human-associated samples. *FEMS Microbiol. Lett.* 307 80–86. 10.1111/j.1574-6968.2010.01965.x 20412303 PMC3148093

[B57] León M. J. Hoffmann T. Sánchez-Porro C. Heider J. Ventosa A. Bremer E. (2018). Compatible solute synthesis and import by the moderate halophile *Spiribacter salinus*: Physiology and Genomics. *Front. Microbiol.* 9:108. 10.3389/fmicb.2018.00108 29497403 PMC5818414

[B58] Letcher P. M. Vélez C. G. Schultz S. Powell M. J. (2012). New taxa are delineated in Alphamycetaceae (Rhizophydiales, Chytridiomycota). *Nova Hedwigia* 94 9–29. 10.1127/0029-5035/2012/0094-0009

[B59] Li J. Li A. M. Li Y. Cai M. H. Luo G. Wu Y. P. (2022). PICRUSt2 functionally predicts organic compounds degradation and sulfate reduction pathways in an acidogenic bioreactor. *Front. Environ. Sci. Eng.* 16:47. 10.1007/s11783-021-1481-8

[B60] Liu G. Zhang Y. van der Mark E. Magic-Knezev A. Pinto A. van den Bogert B. (2018). Assessing the origin of bacteria in tap water and distribution system in an unchlorinated drinking water system by SourceTracker using microbial community fingerprints. *Water Res.* 138 86–96. 10.1016/j.watres.2018.03.043 29573632

[B61] Lozupone C. Knight R. (2005). UniFrac: A new phylogenetic method for comparing microbial communities. *Appl. Environ. Microbiol.* 71 8228–8235. 10.1128/AEM.71.12.8228-8235.2005 16332807 PMC1317376

[B62] Lozupone C. Lladser M. E. Knights D. Stombaugh J. Knight R. (2011). UniFrac: An effective distance metric for microbial community comparison. *ISME J.* 5 169–172. 10.1038/ismej.2010.133 20827291 PMC3105689

[B63] Maegaard K. Nielsen L. P. Revsbech N. P. (2017). Hydrogen dynamics in cyanobacteria dominated microbial mats measured by novel combined H2/H2S and H2/O2 microsensors. *Front. Microbiol.* 8:2022. 10.3389/fmicb.2017.02022 29093704 PMC5651244

[B64] Marchetta A. Papale M. Rappazzo A. C. Rizzo C. Camacho A. Rochera C. (2023). A deep insight into the diversity of microfungal communities in arctic and antarctic lakes. *J. Fungi* 9:1095. 10.3390/jof9111095 37998900 PMC10672340

[B65] Martin M. (2011). Cutadapt removes adapter sequences from high-throughput sequencing rea ds. *EMBnet J.* 17 10–12. 10.14806/ej.17.1.200

[B66] McCord T. B. Hansen G. B. Matson D. L. Johnson T. V. Crowley J. K. Fanale F. P. (1999). Hydrated salt minerals on Europa’s surface from the Galileo near-infrared mapping spectrometer (NIMS) investigation. *J. Geophys. Res. Planets* 104 11827–11851. 10.1029/1999JE900005

[B67] Melack J. M. Jellison R. Macintyre S. Hollibaugh J. T. (2017). “Mono lake: Plankton dynamics over three decades of meromixis or monomixis,” in *Ecology of meromictic lakes*, eds Gulati R. D. Zadereev E. S. Degermendzhi A. G. (Cham: Springer International Publishing), 325–351.

[B68] Miralles-Robledillo J. M. Bernabeu E. Giani M. Martínez-Serna E. Martínez-Espinosa R. M. Pire C. (2021). Distribution of denitrification among haloarchaea: A comprehensive study. *Microorganisms* 9:1669. 10.3390/microorganisms9081669 34442748 PMC8400030

[B69] Murphy J. F. Simandle E. T. Becker D. E. (2003). Population status and conservation of the black toad, bufo exsul. *Southwestern Nat.* 48 54–60. 10.1894/0038-49092003048<0054:PSACOT>2.0.CO;2 40576425

[B70] Nguyen S. T. T. Vardeh D. P. Nelson T. M. Pearson L. A. Kinsela A. S. Neilan B. A. (2022). Bacterial community structure and metabolic potential in microbialite-forming mats from South Australian saline lakes. *Geobiology* 20 546–559. 10.1111/gbi.12489 35312212 PMC9311741

[B71] Nhung N. T. H. Nguyen X. T. Long V. D. Wei Y. Fujita T. (2022). A review of soil contaminated with dioxins and biodegradation technologies: Current status and future prospects. *Toxics* 10:278. 10.3390/toxics10060278 35736887 PMC9227754

[B72] Nielsen M. Revsbech N. P. Kühl M. (2015). Microsensor measurements of hydrogen gas dynamics in cyanobacterial microbial mats. *Front. Microbiol.* 6:726. 10.3389/fmicb.2015.00726 26257714 PMC4508582

[B73] Nosalova L. Mekadim C. Mrazek J. Pristas P. (2023). Thiothrix and Sulfurovum genera dominate bacterial mats in Slovak cold sulfur springs. *Environ. Microbiome* 18:72. 10.1186/s40793-023-00527-4 37730677 PMC10512639

[B74] O’Dea C. Zhang Q. Staley C. Masters N. Kuballa A. Fisher P. (2019). Compositional and temporal stability of fecal taxon libraries for use with SourceTracker in sub-tropical catchments. *Water Res.* 165:114967. 10.1016/j.watres.2019.114967 31430652

[B75] Oremland R. S. (2021). “Salty, alkali-laced tales (Mostly True) from the great basin desert, California and Nevada,” in *Microbes: The foundation stone of the biosphere*, ed. Hurst C. J. (Cham: Springer International Publishing), 653–685.

[B76] Oren A. (2014). The ecology of Dunaliella in high-salt environments. *J. Biol. Res.* 21:23. 10.1186/s40709-014-0023-y 25984505 PMC4389652

[B77] Paquette A. J. Bhatnagar S. Vadlamani A. Gillis T. Khot V. Novotnik B. (2024). Ecology and biogeochemistry of the microbial underworld in two sister soda lakes. *Environ. Microbiome* 19:98. 10.1186/s40793-024-00632-y 39609930 PMC11606062

[B78] Parks D. H. Tyson G. W. Hugenholtz P. Beiko R. G. (2014). STAM: Pstatistical analysis of taxonomic and functional profiles. *Bioinformatics* 30 3123–3124. 10.1093/bioinformatics/btu494 25061070 PMC4609014

[B79] Poser A. Lohmayer R. Vogt C. Knoeller K. Planer-Friedrich B. Sorokin D. (2013). Disproportionation of elemental sulfur by haloalkaliphilic bacteria from soda lakes. *Extremophiles* 17 1003–1012. 10.1007/s00792-013-0582-0 24030483

[B80] Quast C. Pruesse E. Yilmaz P. Gerken J. Schweer T. Yarza P. (2013). The SILVA ribosomal RNA gene database project: Improved data processing and web-based tools. *Nucleic Acids Res.* 41 D590–D596. 10.1093/nar/gks1219 23193283 PMC3531112

[B81] Reigstad L. J. Jorgensen S. L. Lauritzen S. E. Schleper C. Urich T. (2011). Sulfur-oxidizing chemolithotrophic proteobacteria dominate the microbiota in high arctic thermal springs on Svalbard. *Astrobiology* 11 665–678. 10.1089/ast.2010.0551 21899440

[B82] Rubin B. E. Gibbons S. M. Kennedy S. Hampton-Marcell J. Owens S. Gilbert J. A. (2013). Investigating the impact of storage conditions on microbial community composition in soil samples. *PLoS One* 8:e70460. 10.1371/journal.pone.0070460 23936206 PMC3729949

[B83] Sainz-Escudero L. López-Estrada E. K. Rodríguez-Flores P. C. García-París M. (2021). Settling taxonomic and nomenclatural problems in brine shrimps, artemia (Crustacea: branchiopoda: Anostraca), by integrating mitogenomics, marker discordances and nomenclature rules. *PeerJ* 9:e10865. 10.7717/peerj.10865 33854829 PMC7955675

[B84] Sarwa N. Kumari P. Meena D. Udawat P. Chaudhary N. (2024). Alkaline proteases from Haloalkaliphiles: Unveiling Nature’s catalysts for diverse applications. *Appl. Biochem. Microbiol.* 60 855–870. 10.1134/S0003683824603676

[B85] Schindelin J. Arganda-Carreras I. Frise E. Kaynig V. Longair M. Pietzsch T. (2012). Fiji: an open-source platform for biological-image analysis. *Nat. Methods* 2012 9, 676–682. 10.1038/nmeth.2019 22743772 PMC3855844

[B86] Sha C. Wu J. Shen C. Wu J. Yan Z. Wang M. (2023). The ecology of bacterial communities in groundwater of industrial areas: Diversity, composition, network, and assembly. *Environ. Pollut.* 322:121207. 10.1016/j.envpol.2023.121207 36738877

[B87] Sorokin D. Y. Banciu H. L. Muyzer G. (2015a). Functional microbiology of soda lakes. *Curr. Opin. Microbiol.* 25 88–96. 10.1016/j.mib.2015.05.004 26025021

[B88] Sorokin D. Y. Chernyh N. A. Poroshina M. N. (2015b). *Desulfonatronobacter acetoxydans* sp. nov.,: A first acetate-oxidizing, extremely salt-tolerant alkaliphilic SRB from a hypersaline soda lake. *Extremophiles* 19 899–907. 10.1007/s00792-015-0765-y 26085472 PMC4546703

[B89] Sorokin D. Y. Berben T. Melton E. D. Overmars L. Vavourakis C. D. Muyzer G. (2014). Microbial diversity and biogeochemical cycling in soda lakes. *Extremophiles* 18 791–809. 10.1007/s00792-014-0670-9 25156418 PMC4158274

[B90] Sorokin D. Y. Detkova E. N. Muyzer G. (2011a). Sulfur-dependent respiration under extremely haloalkaline conditions in soda lake ‘acetogens’ and the description of *Natroniella sulfidigena* sp. nov. *FEMS Microbiol. Lett.* 319 88–95. 10.1111/j.1574-6968.2011.02272.x 21438913

[B91] Sorokin D. Y. Kuenen J. G. Muyzer G. (2011b). The microbial sulfur cycle at extremely haloalkaline conditions of soda lakes. *Front. Microbiol.* 2:44. 10.3389/fmicb.2011.00044 21747784 PMC3128939

[B92] Sorokin D. Y. Tourova T. P. Kolganova T. V. Detkova E. N. Galinski E. A. Muyzer G. (2011c). Culturable diversity of lithotrophic haloalkaliphilic sulfate-reducing bacteria in soda lakes and the description of *Desulfonatronum thioautotrophicum* sp. nov. *Extremophiles* 15 391–401. 10.1007/s00792-011-0370-7 21479878 PMC3084936

[B93] Sorokin D. Y. Kuenen J. G. (2005a). Chemolithotrophic haloalkaliphiles from soda lakes. *FEMS Microbiol. Ecol.* 52 287–295. 10.1016/j.femsec.2005.02.012 16329914

[B94] Sorokin D. Y. Kuenen J. G. (2005b). Haloalkaliphilic sulfur-oxidizing bacteria in soda lakes. *FEMS Microbiol. Rev.* 29 685–702. 10.1016/j.femsre.2004.10.005 16102598

[B95] Sorokin D. Y. Mosier D. Zorz J. K. Dong X. Strous M. (2020). Wenzhouxiangella strain AB-CW3, a proteolytic bacterium from hypersaline soda lakes that preys on cells of gram-positive bacteria. *Front. Microbiol.* 11:597686. 10.3389/fmicb.2020.597686 33281797 PMC7691419

[B96] Sorokin D. Y. Tikhonova T. V. Koch H. van den Berg E. M. Hinderks R. S. Pabst M. (2023). Trichlorobacter ammonificans, a dedicated acetate-dependent ammonifier with a novel module for dissimilatory nitrate reduction to ammonia. *ISME J.* 17 1639–1648. 10.1038/s41396-023-01473-2 37443340 PMC10504241

[B97] Sorokin D. Y. Yakimov M. Messina E. Merkel A. Y. Bale N. J. Sinninghe Damsté J. S. (2019). *Natronolimnobius sulfurireducens* sp. nov. and *Halalkaliarchaeum desulfuricum* gen. nov., sp. nov., the first sulfur-respiring alkaliphilic haloarchaea from hypersaline alkaline lakes. *Int. J. Syst. Evol. Microbiol.* 69 2662–2673. 10.1099/ijsem.0.003506 31166158

[B98] Soufi H. H. Tran D. Louca S. (2024). Microbiology of Big Soda Lake, a multi-extreme meromictic volcanic crater lake in the Nevada desert. *Environ. Microbiol.* 26:e16578. 10.1111/1462-2920.16578 38350645

[B99] Staley C. Kaiser T. Lobos A. Ahmed W. Harwood V. J. Brown C. M. (2018). Application of sourcetracker for accurate identification of fecal pollution in recreational freshwater: A double-blinded study. *Environ. Sci. Technol.* 52 4207–4217. 10.1021/acs.est.7b05401 29505249

[B100] Stamps B. W. Nunn H. S. Petryshyn V. A. Oremland R. S. Miller L. G. Rosen M. R. (2018). Metabolic capability and phylogenetic diversity of mono lake during a bloom of the eukaryotic phototroph *Picocystis sp*. Strain ML. *Appl. Environ. Microbiol.* 84:e01171-18. 10.1128/AEM.01171-18 30120120 PMC6193381

[B101] Steinbach R. M. El Baidouri F. Vogt E. Mitchison-Field L. M. Y. Lim F. Y. Ekena J. (2023). Malassezia is widespread and has undescribed diversity in the marine environment. *Fungal Ecol.* 65:101273. 10.1016/j.funeco.2023.101273 40893143 PMC12392166

[B102] Tabares M. Kashefi K. Reguera G. (2025). Adaptive responses of Trichlorobacter lovleyi to nitrite detoxification reveal overlooked contributions of Geobacterales to nitrate ammonification. *ISME J.* 19:wraf054. 10.1093/ismejo/wraf054 40101204 PMC11972089

[B103] Tang Y. Huo J. Zhu D. Yuan Z. (2022). Simulation of the water storage capacity of siling co lake on the tibetan plateau and its hydrological response to climate change. *Water* 14:3175. 10.3390/w14193175

[B104] Teunisse G. M. (2022). *Fantaxtic - Nested bar plots for phyloseq data.* San Francisco, CA: github.com.

[B105] The Galaxy Community (2024). The Galaxy platform for accessible, reproducible, and collaborative data analyses: 2024 update. *Nucleic Acids Res.* 52 W83–W94. 10.1093/nar/gkae410 38769056 PMC11223835

[B106] Tomonaga Y. Brennwald M. S. Livingstone D. M. Kwiecien O. Randlett M. È Stockhecke M. (2017). Porewater salinity reveals past lake-level changes in Lake Van, the Earth’s largest soda lake. *Sci. Rep.* 7:313. 10.1038/s41598-017-00371-w 28331216 PMC5428207

[B107] Toner J. D. Catling D. C. (2020). A carbonate-rich lake solution to the phosphate problem of the origin of life. *Proc. Natl. Acad. Sci. U. S. A.* 117 883–888. 10.1073/pnas.1916109117 31888981 PMC6969521

[B108] Toole D. R. Zhao J. Martens-Habbena W. Strauss S. L. (2021). Bacterial functional prediction tools detect but underestimate metabolic diversity compared to shotgun metagenomics in southwest Florida soils. *Appl. Soil Ecol.* 168:104129. 10.1016/j.apsoil.2021.104129

[B109] Uma G. Babu M. M. Prakash V. S. G. Nisha S. J. Citarasu T. (2020). Nature and bioprospecting of haloalkaliphilics: A review. *World J. Microbiol. Biotechnol.* 36:66. 10.1007/s11274-020-02841-2 32323057

[B110] Usyk M. Zolnik C. P. Patel H. Levi M. H. Burk R. D. (2017). Novel ITS1 fungal primers for characterization of the mycobiome. *mSphere* 2:e00488-17. 10.1128/mSphere.00488-17 29242834 PMC5729218

[B111] Valderrama J. A. Durante-Rodríguez G. Blázquez B. García J. L. Carmona M. Díaz E. (2012). Bacterial degradation of benzoate: Cross-regulation between aerobic and anaerobic pathways. *J. Biol. Chem.* 287 10494–10508. 10.1074/jbc.M111.309005 22303008 PMC3322966

[B112] Varshney S. Bhattacharya A. Gupta A. (2023). Halo-alkaliphilic microbes as an effective tool for heavy metal pollution abatement and resource recovery: Challenges and future prospects. *3 Biotech* 13:400. 10.1007/s13205-023-03807-5 37982082 PMC10651602

[B113] Vavourakis C. D. Mehrshad M. Balkema C. van Hall R. Andrei A. S. Ghai R. (2019). Metagenomes and metatranscriptomes shed new light on the microbial-mediated sulfur cycle in a Siberian soda lake. *BMC Biol.* 17:69. 10.1186/s12915-019-0688-7 31438955 PMC6704655

[B114] Vázquez-Baeza Y. Pirrung M. Gonzalez A. Knight R. (2013). EMPeror: A tool for visualizing high-throughput microbial community data. *Gigascience* 2:16. 10.1186/2047-217X-2-16 24280061 PMC4076506

[B115] Velegraki A. Cafarchia C. Gaitanis G. Iatta R. Boekhout T. (2015). Malassezia infections in humans and animals: Pathophysiology, detection, and treatment. *PLoS Pathog* 11:e1004523. 10.1371/journal.ppat.1004523 25569140 PMC4287564

[B116] Vera A. Wilson F. P. Cupples A. M. (2022). Predicted functional genes for the biodegradation of xenobiotics in groundwater and sediment at two contaminated naval sites. *Appl. Microbiol. Biotechnol.* 106 835–853. 10.1007/s00253-021-11756-3 35015144

[B117] Viana A. T. Caetano T. Covas C. Santos T. Mendo S. (2018). Environmental superbugs: The case study of *Pedobacter spp*. *Environ. Pollut.* 241 1048–1055. 10.1016/j.envpol.2018.06.047 30029312

[B118] Walters W. Hyde E. R. Berg-Lyons D. Ackermann G. Humphrey G. Parada A. (2016). Improved bacterial 16S rRNA Gene (V4 and V4-5) and fungal internal transcribed spacer marker gene primers for microbial community surveys. *mSystems* 1:e00009-15. 10.1128/mSystems.00009-15 27822518 PMC5069754

[B119] Wang I. J. (2009). Fine-scale population structure in a desert amphibian: Landscape genetics of the black toad (Bufo exsul). *Mol. Ecol.* 18 3847–3856. 10.1111/j.1365-294X.2009.04338.x 19708887

[B120] Western Regional Climate Center [WRCC]. Available online at: https://wrcc.dri.edu/cgi-bin/cliMAIN.pl?cadeep+nca.

[B121] Wright R. J. Langille M. G. I. (2025). PICRUSt2-SC: An update to the reference database used for functional prediction within PICRUSt2. *Bioinformatics* 41:btaf269. 10.1093/bioinformatics/btaf269 40293718 PMC12089645

[B122] Wu Y. T. Chiang P. W. Tandon K. Rogozin D. Y. Degermendzhy A. G. Tang S. L. (2021). Single-cell genomics-based analysis reveals a vital ecological role of *Thiocapsa sp*. LSW in the meromictic Lake Shunet, Siberia. *Microb Genom.* 7:000712. 10.1099/mgen.0.000712 34860152 PMC8767323

[B123] Xue Q. Zhao D. Zhang S. Zhou H. Zuo Z. Zhou J. (2021). Highly integrated adaptive mechanisms in *Spiribacter halalkaliphilus*, a bacterium abundant in Chinese soda-saline lakes. *Environ. Microbiol.* 23 6463–6482. 10.1111/1462-2920.15794 34587356 PMC9292931

[B124] Young J. Holland I. B. (1999). ABC transporters: Bacterial exporters-revisited five years on. *Biochim. Biophys. Acta* 1461 177–200. 10.1016/s0005-2736(99)00158-3 10581355

[B125] Zipkin E. F. DiRenzo G. V. (2022). Biodiversity is decimated by the cascading effects of the amphibian-killing chytrid fungus. *PLoS Pathog* 18:e1010624. 10.1371/journal.ppat.1010624 35862362 PMC9302726

[B126] Zorz J. K. Sharp C. Kleiner M. Gordon P. M. K. Pon R. T. Dong X. (2019). A shared core microbiome in soda lakes separated by large distances. *Nat. Commun.* 10:4230. 10.1038/s41467-019-12195-5 31530813 PMC6748926

