# Supplementary material for: Microbial communities of selected regions of the Deep Springs Lake aquifer system
Source: Front Microbiol. 2025 Dec 18;16:1689006. doi: 10.3389/fmicb.2025.1689006 (PMC12756364; doi:10.3389/fmicb.2025.1689006)
Supplement: Supplementary file 1 [file Data_Sheet_1.pdf]

Supplementary Material

Microbial Communities of Selected Regions of the Deep Springs Lake Aquifer System

1 Maps and Samples

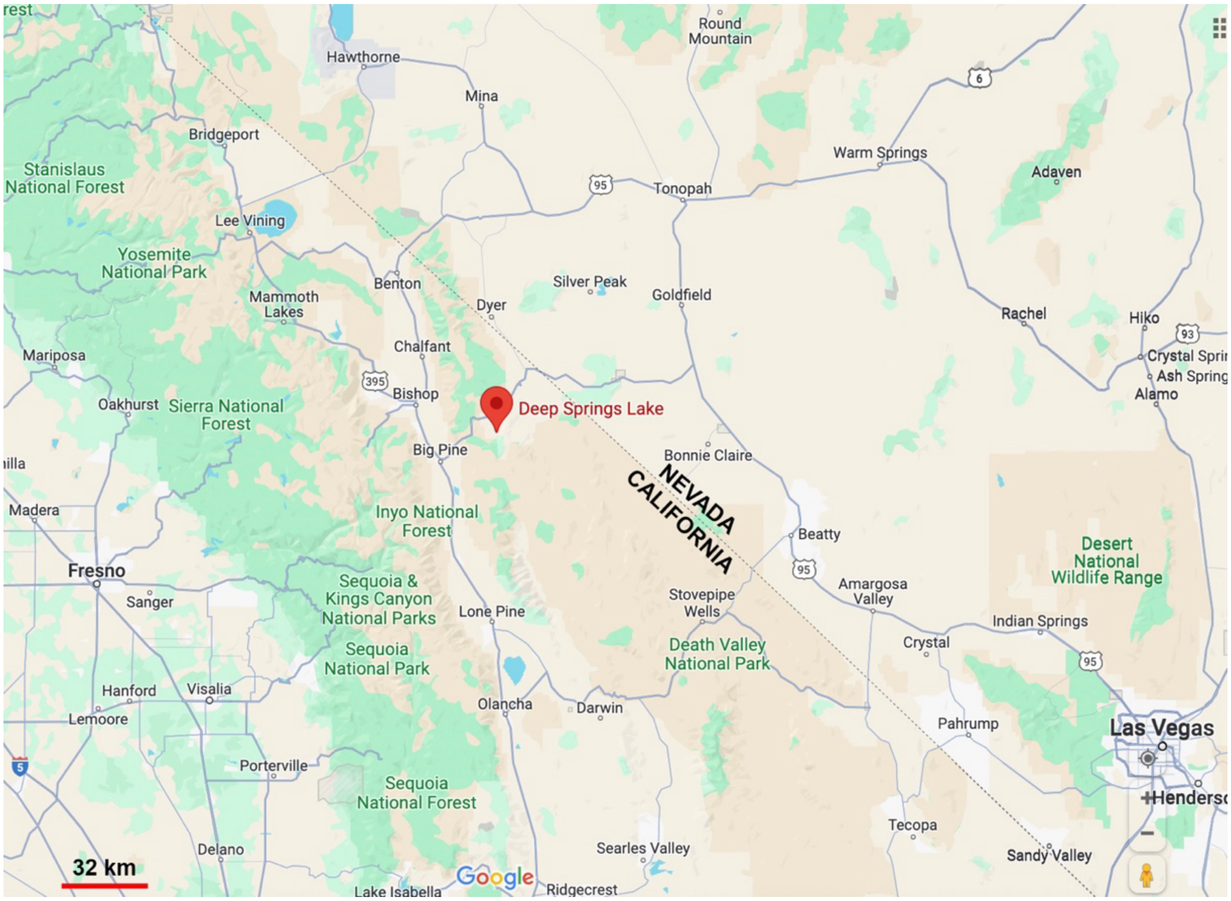

Figure S1. Context map of the Deep Springs Like area showing the Nevada/California state line and several major cities and national parks. Image: Google Maps.

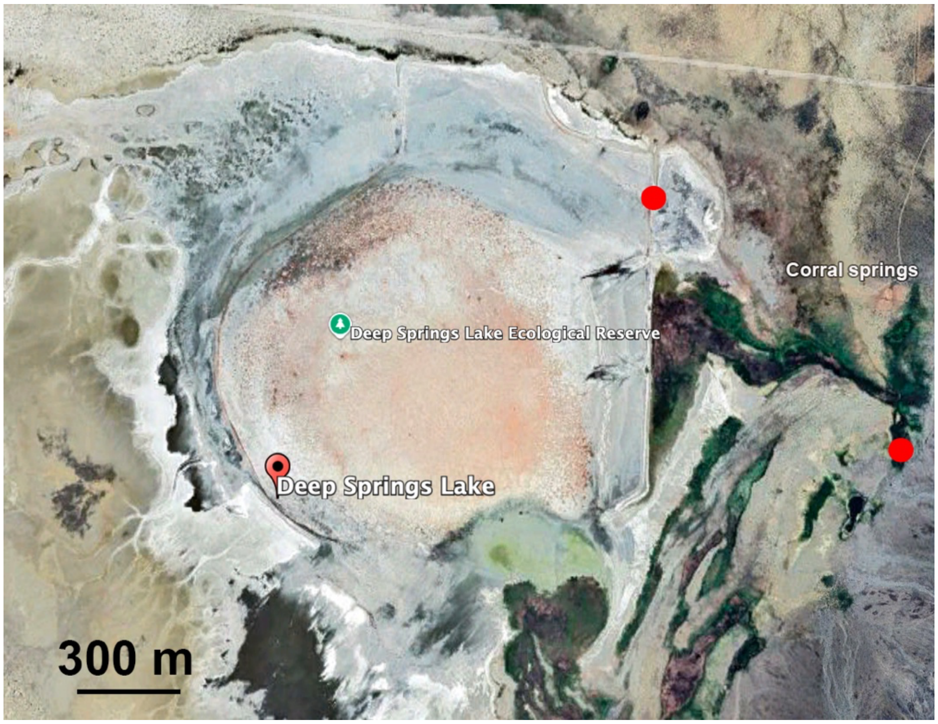

Figure S2. Sampling sites for lake water and salt crust (upper red circle) and Corral Springs biofilm (lower red circle). Map source: Google Earth.

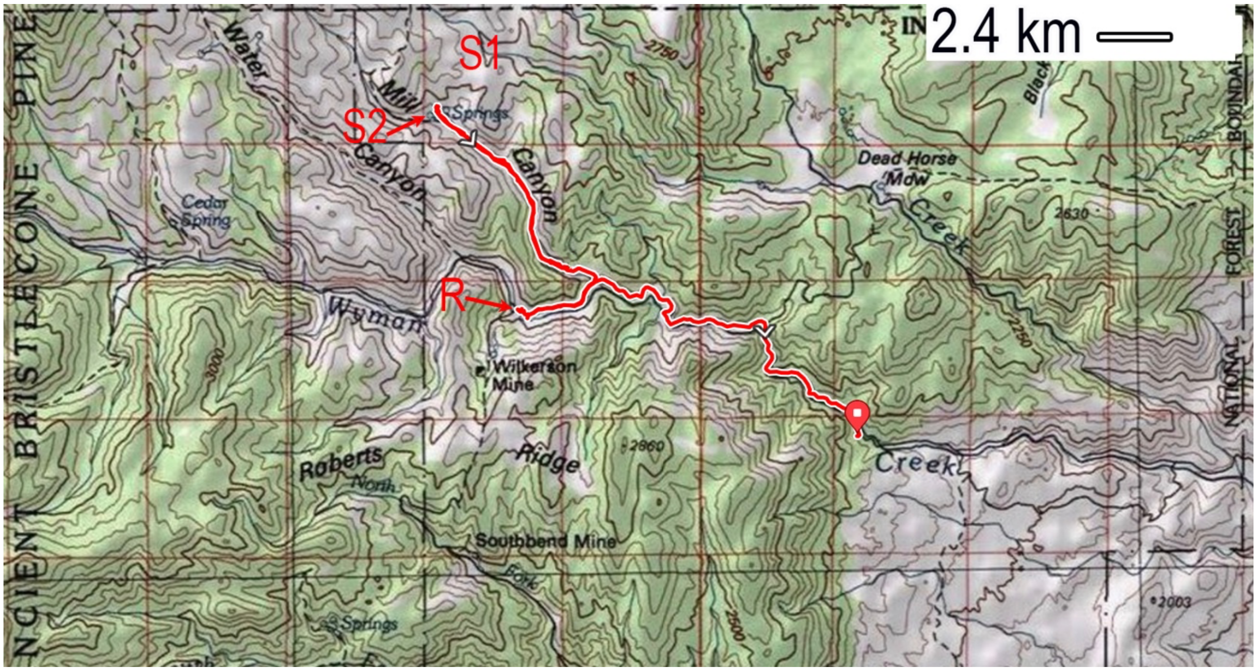

Figure S3. USGS topographical map of area between Wyman Creek/Roberts Ranch Stream (RS) and Springs 1 and 2 (S1, S2). Note that the springs feature on the map.

Sample name	Sample abbreviation	Collection date	Lat./Long.	Mass/volume of collection	Purpose	Description	Stat Group
Lake water 1	LW1	03/20/24	37 º16'50.63" N, 118 º01'54.11"W	4 L	Sequencing, imaging, trace mineral analysis	Open water from edge of lake sampled by filling 2L bottles	A
Lake water 2	LW2	03/20/24	37 º16'50.63" N, 118 º01'54.11"W	2 L	Sequencing, imaging	Replicate lake water sample	A
Lake water 3	ON	03/20/24	37 º16'50.63" N, 118 º01'54.11"W	2 L	Sequencing, imaging	Replicate lake water sample used for overnight incubation	A
Corral Springs Biofilm 1	BF1	03/20/24	37 º16'26.29" N, 118 º01'23.51"W	100 mL	Sequencing, imaging, trace mineral analysis	Green microbial mat and surrounding water	B
Corral Springs Biofilm 2	BF2	03/20/24	37 º16'26.29" N, 118 º01'23.51"W	100 mL	Sequencing, imaging	Replicate biofilm sample	B
Salt crust	Salt	03/20/24	37 º16'50.63" N, 118 º01'54.11"W	200 g	Sequencing, imaging	Salt crust immediately outside the standing water of the lake	B
Spring 1	S1	04/03/24	37º27'10.04"N, 118º 06' 40.36"W	2 L	Sequencing, imaging, trace mineral analysis	Spring water sampled as described in text	C
Spring 2	S2	04/03/24	37º27'9.9"N, 118º 06' 41.51"W	2 L	Sequencing, imaging, trace mineral analysis	Spring water sampled as described in text	C
Roberts Ranch Spring	RS	04/03/24	37º25'51.02"N, 118º 05' 58.02"W	2 L	Sequencing, imaging, trace mineral analysis	Spring water sampled as described in text	C

Table S1. Samples collected for this study. The abbreviations indicate those used in figure captions throughout the paper. "Stat Group" indicates which samples were combined for statistical analyses of diversity.

2 Sequencing Methods and Denoising

2.1 Methods provided by Molecular Research (www.mrdnalab.com)

The selected primers were used in a 30 cycle PCR using the HotStarTaq Plus Master Mix Kit (Qiagen) under the following conditions: 95°C for 5 minutes, followed by 30-35 cycles of 95°C for 30 seconds, 53°C for 40 seconds and 72°C for 1 minute, after which a final elongation step at 72°C for 10 minutes was performed. After amplification, PCR products are checked in 2% agarose gel to determine the success of amplification and the relative intensity of bands. Samples are multiplexed

using unique dual indices and are pooled together in equal proportions based on their molecular weight and DNA concentrations. Pooled samples are purified using calibrated Ampure XP beads. Then the pooled and purified PCR product is used to prepare an Illumina DNA library. Sequencing was performed on a MiSeq following the manufacturer’s guidelines. Primers used were: Bacteria, 16S rRNA gene V4 variable region PCR primers 515/806; fungi, ITS1F/ITS2 primer pair; Eukaryotes, 18S rRNA gene 1391f.

2.2 Denoising data, bacteria

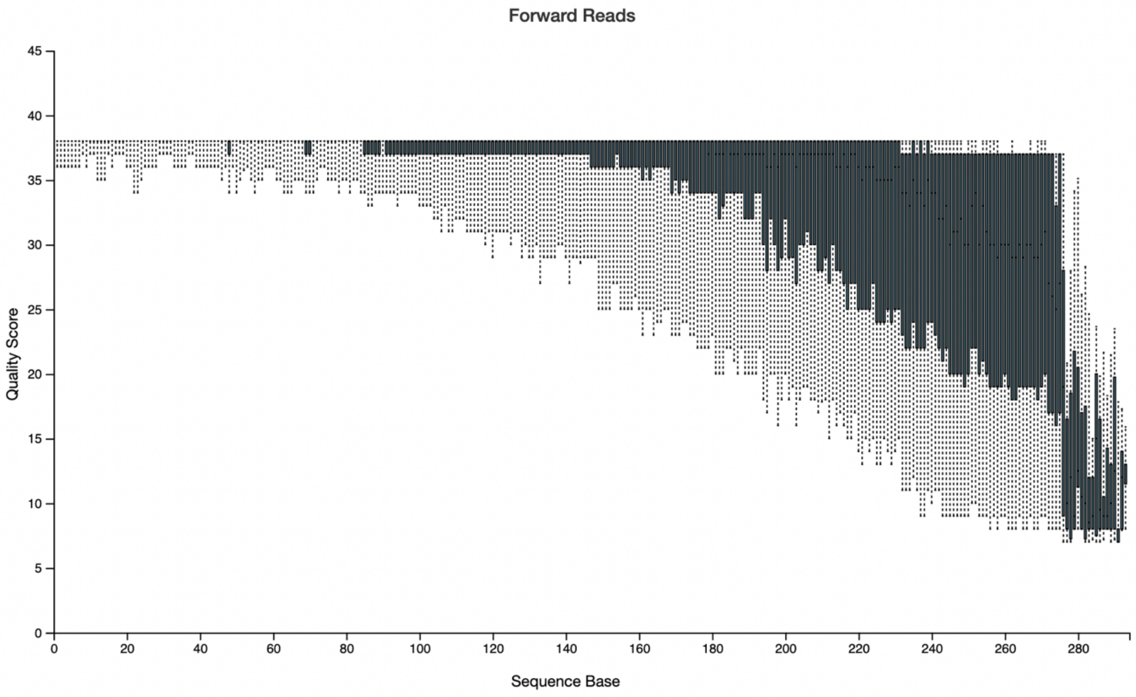

Figure S4. Interactive quality plot for forward reads, 16S.

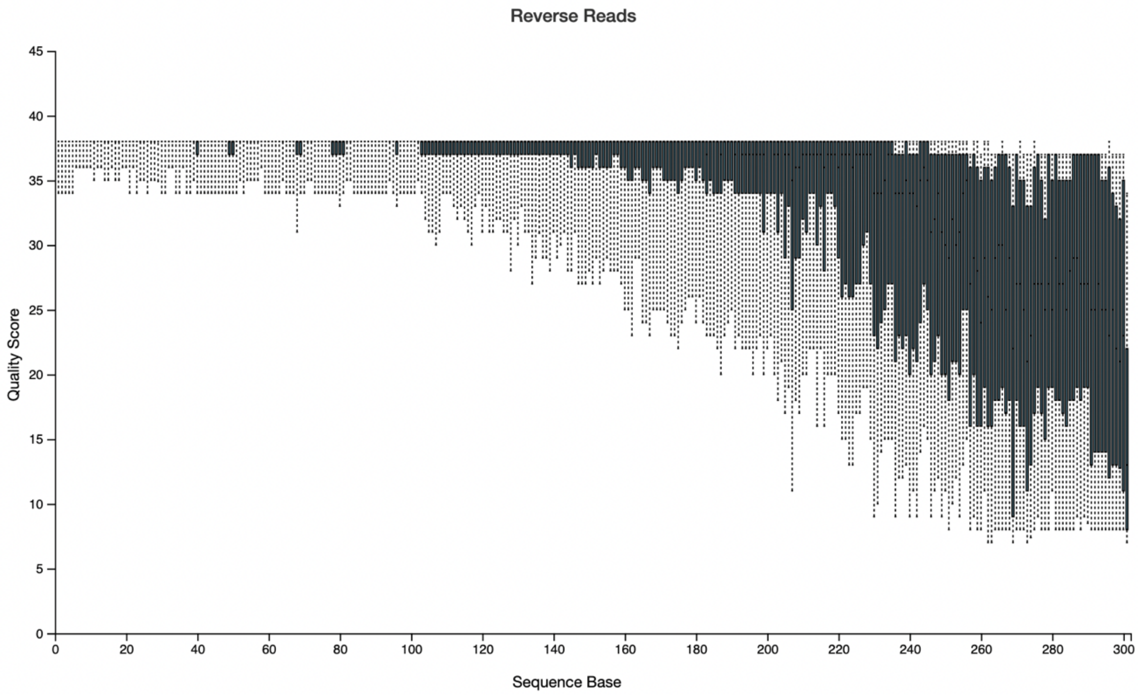

Figure S5. Interactive quality plot for reverse reads, 16S.

sample ID	forward sequence count	reverse sequence count
**Spring.1**	101981	101981
**Spring.2**	97155	97155
**Salt**	83987	83987
**Roberts.Spring**	80100	80100
**Overnight.ON**	79903	79903
**Lake.water.1.LW1**	75611	75611
**Lake.water.2.LW2**	73442	73442
**Biofilm.1.BF1**	69830	69830
**Biofilm.2.BF2**	62598	62598

Table S2. Read counts for 16S.

sample-id	input	filtered	%passed filter	denoised	merged	%merged	non-chimeric	%non-chimeric
Biofilm.1.BF1	69830	53829	77.09	49076	41349	59.21	39775	56.96
Biofilm.2.BF2	62598	47680	76.17	43568	37001	59.11	35838	57.25
Lake.water.1.LW1	75611	58130	76.88	53970	44566	58.94	42038	55.6
Lake.water.2.LW2	73442	56476	76.9	52607	43155	58.76	40412	55.03
Overnight.ON	79903	62245	77.9	60184	52967	66.29	43141	53.99
Roberts.Spring	80100	47521	59.33	39199	24765	30.92	22362	27.92
Salt	83987	64434	76.72	59197	51035	60.77	46799	55.72
Spring.1	101981	58543	57.41	52144	34431	33.76	34242	33.58
Spring.2	97155	60066	61.82	49898	31942	32.88	30480	31.37

Table S3. Denoising stats for 16S using the following parameters optimized for merging: trunc_len_f :270, trunc_len_r: 280, max_ee_f: 3.0, max_ee_r: 6.0, min_overlap: 12.

2.3 Denoising data, fungi

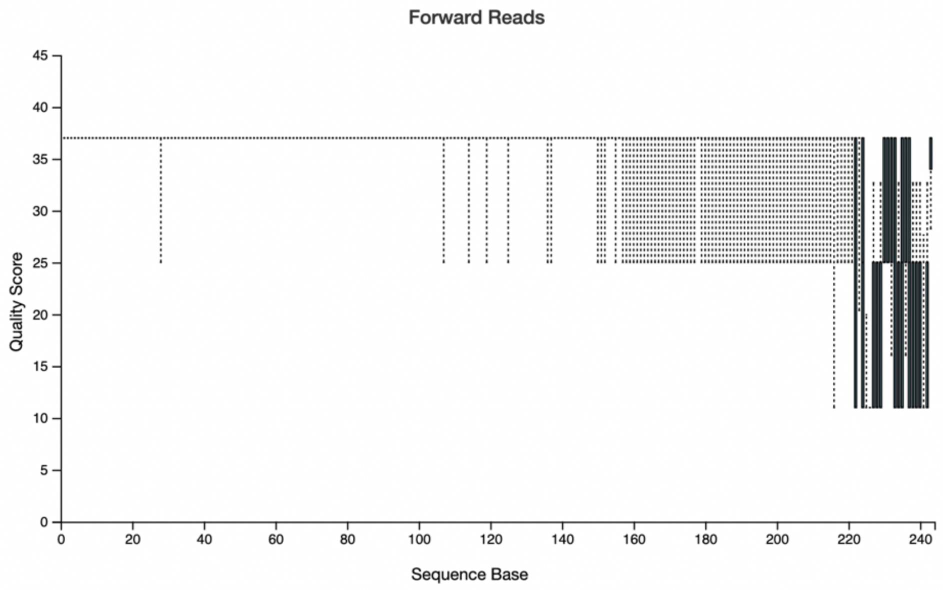

Figure S6. Interactive quality plot for forward reads, ITS.

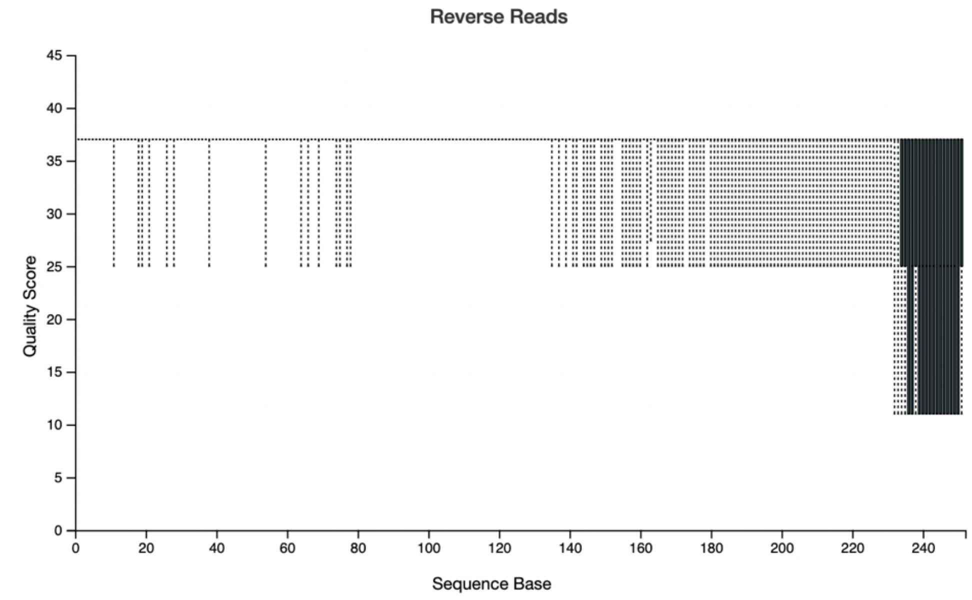

Figure S7. Interactive quality plot for reverse reads, ITS.

sample ID	forward sequence count	reverse sequence count
Salt.ITS	1406495	1406495
Biofilm.1.BF1.ITS	1343924	1343924
Overnight.ON.ITS	1160168	1160168
Biofilm.2.BF2.ITS	1123485	1123485
Lake.water.1.LW1.ITS	912304	912304
Lake.water.2.LW2.ITS	897529	897529
Blank.ITS	784709	784709

Table S4. Read counts for ITS.

sample-id	input	filtered	percentage	denoised	merged	percentage	non-chimeric	percentage of input non-chimeric
Biofilm.1.BF1.ITS	1343924	1022103	76.05	1018084	861699	64.12	841949	62.65
Biofilm.2.BF2.ITS	1123485	847987	75.48	843964	696167	61.96	683010	60.79
Blank.ITS	784709	672419	85.69	669705	654316	83.38	646752	82.42
Lake.water.1.LW1.ITS	912304	800505	87.75	795738	763096	83.64	744027	81.55
Lake.water.2.LW2.ITS	897529	786918	87.68	783629	751255	83.7	730879	81.43
Overnight.ON.ITS	1160168	1039565	89.6	1035622	994247	85.7	957705	82.55
Salt.ITS	1406495	1127296	80.15	1121701	940409	66.86	913495	64.95

Table S5. Denoising stats for ITS using dada2-denoise-paired with the following parameters: trunc_len_f :0, trunc_len_r: 0, max_ee_f: 3.0, max_ee_r: 6.0, min_overlap: 12

2.4 Eukaryotic Data Analysis

Provided by Molecular Research (www.mrdnalab.com)

The Q25 sequence data derived from the sequencing process was processed using the MR DNA ribosomal and functional gene analysis pipeline (http://www.mrdnalab.com/, MR DNA, Shallowater, TX). Sequences are depleted of primers, short sequences < 150bp are removed, and sequences with ambiguous base calls removed. Sequences are quality filtered using a maximum expected error threshold of 1.0 and dereplicated. The dereplicated or unique sequences are denoised; unique sequences identified with sequencing or PCR point errors are removed, followed by chimera removal, thereby providing a denoised sequence or zOTU. Final zOTUs were taxonomically classified using BLAST against a curated database derived from NCBI (http://www.ncbi.nlm.nih.gov/) and compiled into each taxonomic level into both "counts" and "percentage" files.

3 Hydrology

	**Lake, this study**	**Lake,** Reported June 2019 [1]	**Lake,** Reported 2011 [2]	**Corral Springs ,** Reported 1959 [3]
pH	9.4	9.4	9.4	8.3
Na (mM)		2600	4000	1.74-10
Ca (mM)	0.46	.06	0.30	0.3-1.3
K (mM)	3.2	160	250	0.15-0.45
Mg (mM)	0.92	0.21	0.90	0.1-0.6
SO_4 (mM)		500	200-800	0.4-2.1
Si (mM)		0.5	NR	0.50

	Springs 1, 2 (this study)	Wyman Creek/Roberts Ranch Stream, this study	Wyman Creek below Mill Canyon, Reported 1959 [3]	Roberts Ranch, Reported 1959 [3]
pH	**7.8**	**7.9**	**7.8**	**7.5**
Electrical conductivity (dS/m)	**0.25**	**0.18**		
TDS			**232**	**244**
Na (ppm)			**7.3**	**12**
Ca (ppm)	**53**	**38**	**56**	**53**
K (ppm)	**7.5**	**4.1**	**2.3**	**6**
Mg (ppm)	**3.1**	**7.9**	**11**	**16**
Fe (ppm)	**0.02**	**0.02**		
P (ppm)	**1.4**	**0.7**		
SO_4 (ppm)			**37**	**17**

Si (ppm)			**19**	**20**
Bicarbonate (HCO_2) (ppm)			**198**	**234**
Cl (ppm)			**1.0**	**4.5**

Table S6. Bulk properties and trace minerals of the liquid water samples. Values measured here were compared with those seen in previous studies at comparable locations as referenced. Wyman Creek below Mill Canyon is the closest reported site to Springs 1 and 2. Roberts Ranch is the closest reported site to to the Wyman Creek/Roberts Ranch Stream sample in this study. Dates sampled are given rather than the year that the publication appeared. Values are given in mM for the lake water and ppm for the springs for easier comparison with the references cited. BQL= below quantifiable limits. Zn and Mn were both BQL for all samples measured here (<0.5 ppm for Zn, <1.0 ppm for Mn). The values from Springs 1 and 2 were measured separately but did not differ to our level of sensitivity, so are reported together.

(1) Hobbs, F. W. C.; Fang, Y.; Lebrun, N.; Yang, Y.; Xu, H. Co-precipitation of primary dolomite and Mg-rich clays in Deep Springs Lake, California. *Sedimentology* **2024**, *71* (4), 1363-1383. DOI: https://doi.org/10.1111/sed.13176 (acccessed 2024/08/08).

(2) Meister, P.; Reyes, C.; Beaumont, W.; Rincon, M.; Collins, L.; Berelson, W.; Stott, L.; Corsetti, F.; Nealson, K. H. Calcium and magnesium-limited dolomite precipitation at Deep Springs Lake, California. *Sedimentology* **2011**, *58* (7), 1810-1830. DOI: https://doi.org/10.1111/j.1365-3091.2011.01240.x (acccessed 2025/06/10).

(3) Jones, B. F. *The hydrology and mineralogy of Deep Springs Lake, Inyo County, California*; 1965. https://pubs.usgs.gov/publication/pp502ADOI: 10.3133/pp502A.

4 Diversity metrics

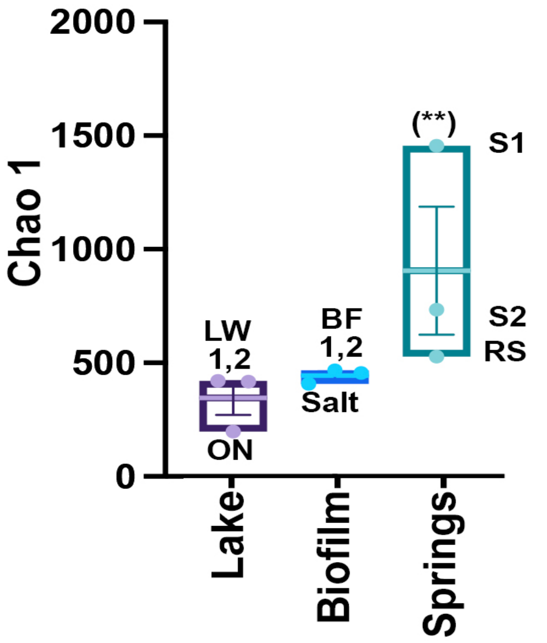

Figure S8. Chao1 Alpha diversity by site, n=3 for each, with individual sites noted. The error bars indicate standard error of the mean and the boxes indicate high and low values. (**) indicates $p<0.05$ for comparison with other groups.

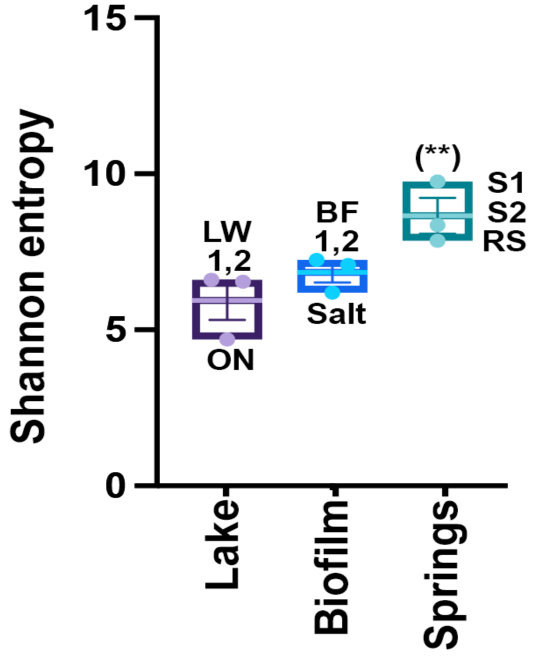

Figure S9. Shannon entropy by site, n=3 for each, with individual sites noted. The error bars indicate standard error of the mean and the boxes indicate high and low values. (**) indicates $p<0.05$ for comparison with other groups.

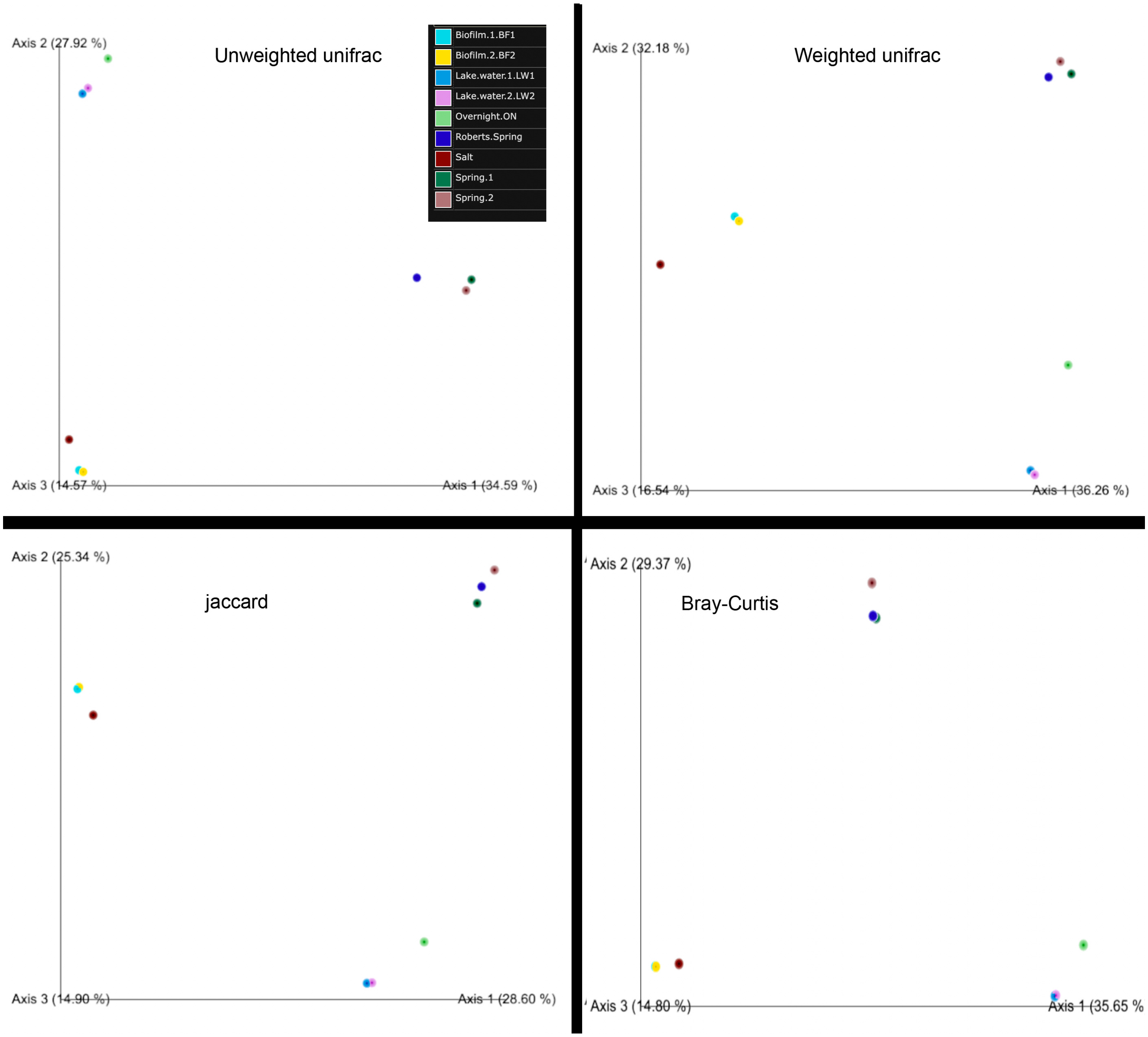

Figure S10. Beta diversity plots showing data for each individual sites. Metrics shown are unweighted UniFrac (1), weighted UniFrac (2), Jaccard, and Bray-Curtis dissimilarity. Note that the Biofilm 1 and Biofilm 2 samples nearly overlap in the Jaccard and Bray-Curtis plots.

(1) Lozupone C, Knight R. UniFrac: a new phylogenetic method for comparing microbial communities. Appl Environ Microbiol. 2005;71:8228-8235.
(2) Lozupone CA, Hamady M, Kelley ST, et al. Quantitative and qualitative beta diversity measures lead to different insights into factors that structure microbial communities. Appl Environ Microbiol. 2007;73:1576-1585.

5 Abundances

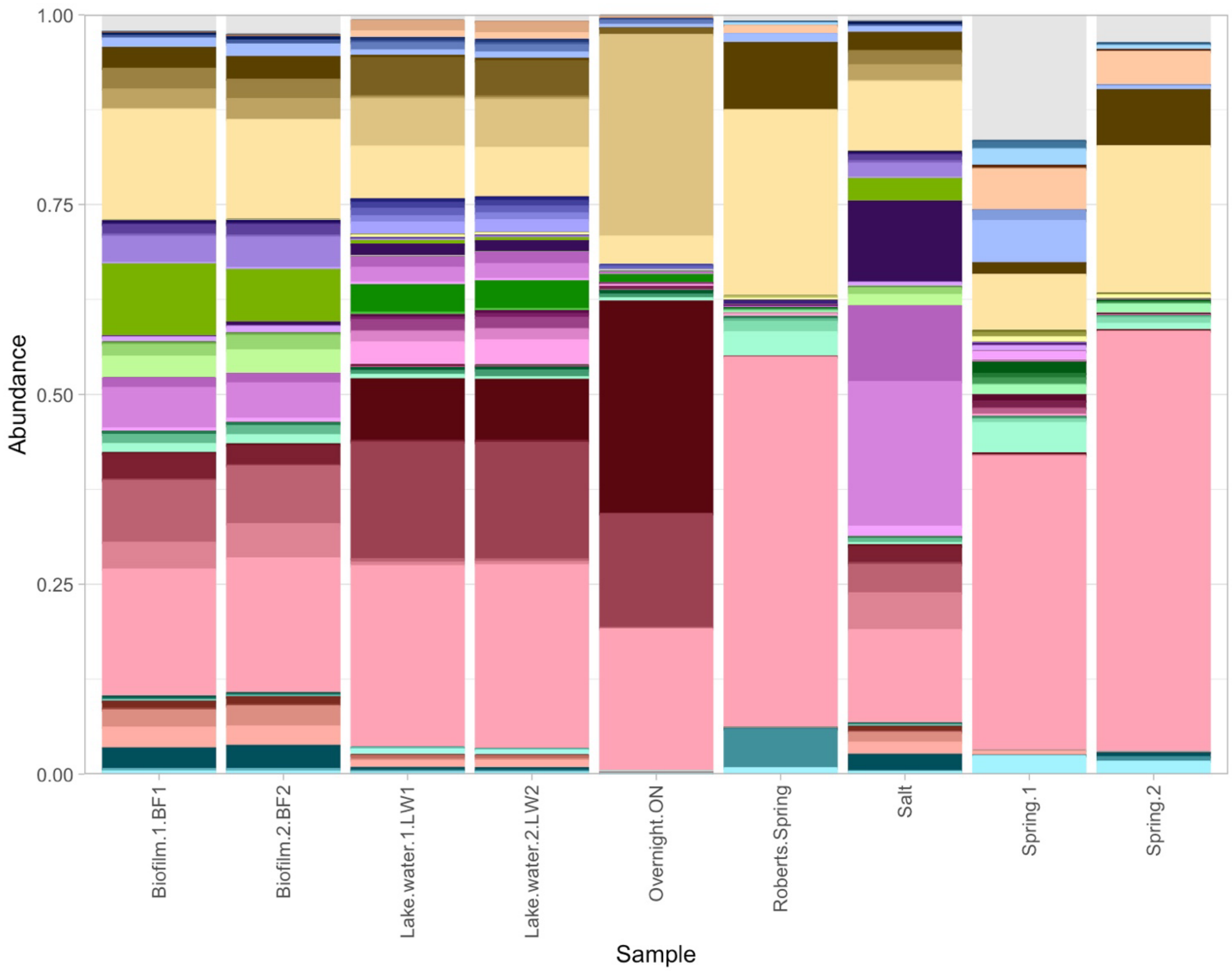

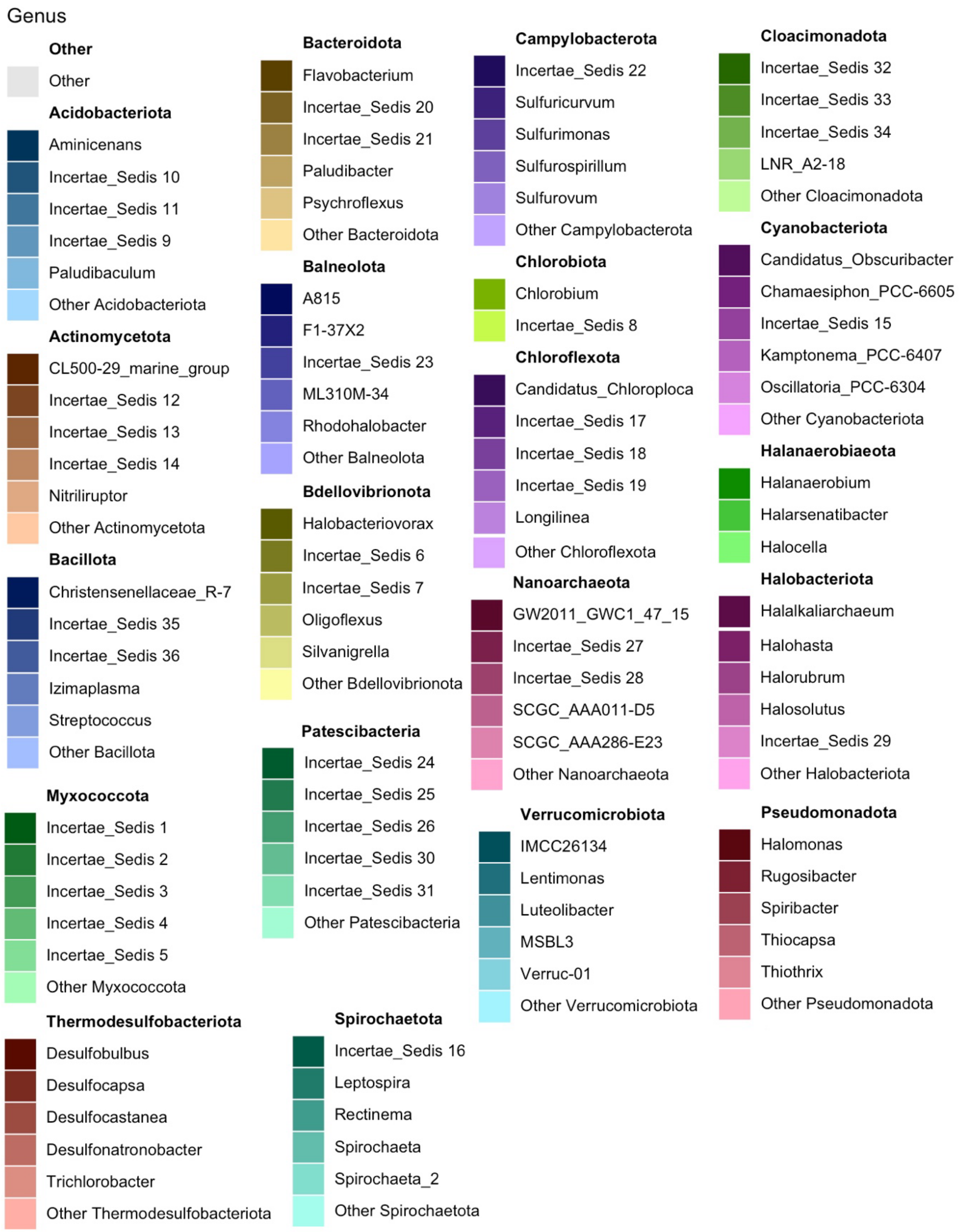

Figure S11. Prokaryotic abundances by genus, color-coded by phylum, for the top 20 phyla and 5 genera overall.

sample	weighted_NSTI
**Biofilm.1.BF1**	0.177
**Biofilm.2.BF2**	0.180
**Blank**	0.092
**Lake.water.1.LW1**	0.174
**Lake.water.2.LW2**	0.175
**Overnight.ON**	0.143
**Salt**	0.122

Table S7. Weighted NSTI scores calculated in Picrust2.

g__Pleosporales
g__Myriangiales
p__Fungi_phy_Incertae_sedis
g__Cladosporium
g__Udeniomyces
g__Naganishia
g__Filobasidium
g__Aspergillus
g__Herpotrichiellaceae
g__Vishniacozyma
g__Alternaria
g__Candida
g__Pleurotus
g__Agaricaceae
g__Symmetrospora
g__Zasmidium
g__Cystofilobasidium
g__Podosphaera
g__Phaeococcomyces
g__Chaetosphaeronema
g__Gelidatrema
g__Trichomerium
g__Aureobasidium
g__Neoascochyta
f__Phanerochaetaceae
g__Debaryomyces
g__Endoconidioma
g__Wallemia

Table S7. Fungi occurring at the highest frequency in the blank sample and removed from analysis.
